# Exposure to stressors and antimicrobials induces cell-autonomous ultrastructural heterogeneity of an intracellular bacterial pathogen

**DOI:** 10.3389/fcimb.2022.963354

**Published:** 2022-11-15

**Authors:** Marc Schulte, Michael Hensel, Katarzyna Miskiewicz

**Affiliations:** Division of Microbiology, and CellNanOs – Center of Cellular Nanoanalytics Osnabrück, Universität Osnabrück, Osnabrück, Germany

**Keywords:** intracellular pathogen, bacterial cytoplasm, CLEM, bacterial heterogeneity, stress response

## Abstract

Despite their clonality, intracellular bacterial pathogens commonly show remarkable physiological heterogeneity during infection of host cells. Physiological heterogeneity results in distinct ultrastructural morphotypes, but the correlation between bacterial physiological state and ultrastructural appearance remains to be established. In this study, we showed that individual cells of *Salmonella enterica* serovar Typhimurium are heterogeneous in their ultrastructure. Two morphotypes based on the criterion of cytoplasmic density were discriminated after growth under standard culture conditions, as well as during intracellular lifestyle in mammalian host cells. We identified environmental conditions which affect cytoplasmic densities. Using compounds generating oxygen radicals and defined mutant strains, we were able to link the occurrence of an electron-dense ultrastructural morphotype to exposure to oxidative stress and other stressors. Furthermore, by combining ultrastructural analyses of *Salmonella* during infection and fluorescence reporter analyses for cell viability, we provided evidence that two characterized ultrastructural morphotypes with electron-lucent or electron-dense cytoplasm represent viable cells. Moreover, the presence of electron-dense types is stress related and can be experimentally induced only when amino acids are available in the medium. Our study proposes ultrastructural morphotypes as marker for physiological states of individual intracellular pathogens providing a new marker for single cell analyses.

## Introduction

Recent research on pathogenic bacteria revealed that cells react independently when exposed to adverse conditions due to differences in their physiological status (Lidstrom and Konopka, 2010; Burton et al., 2014; Claudi et al., 2014; Helaine et al., 2014; Avraham et al., 2015; Khodaparast et al., 2018; Brenzinger et al., 2019). Classification of physiological states and understanding of their biological functions are crucial for designing new antimicrobials, which would overcome bacterial resistance without the risk of imposing selective pressure towards bacterial survival. Despite great progress in understanding the role of individual virulence factors for bacterial pathogenesis, the resilient bacterial survival in detrimental environmental conditions is still enigmatic (LaRock et al., 2015; Fang et al., 2016; Fisher et al., 2017). In the context of stress response, the formation of a dormant state with metabolic shifts, and change in cytoplasmic dynamics was postulated (Konopka et al., 2009; Wood et al., 2019). In general, bacterial cells appear structurally more complex than previously considered. For instance, the bacterial cytoplasm displays, in addition to high molecular crowding, unusual motility dynamics for differently-sized particles, properties of glass-phase, or transitions to solid-states (Golding and Cox, 2006; Parry et al., 2014).

Transmission electron microscopy (TEM) is a potent tool to visualize the composition of bacterial envelopes, protein complexes, and has shed light on protein shell structures, revolutionizing the view on bacterial organelles (Yeates et al., 2010; Mayer et al., 2016). Conventional TEM is broadly used as standard method to evaluate effects of bactericidal compounds (Helander et al., 2001; Hartmann et al., 2010; Yossa et al., 2014; López-Heras et al., 2015; Zhang et al., 2016; Khodaparast et al., 2018). In contrast, TEM has been applied rarely to describe physiological states of pathogenic bacteria at the single cell level.

Bacterial cells visualized by TEM demonstrate a high degree of morphological heterogeneity (Via et al., 1997; Szenasi et al., 1998; Inglis et al., 2000; Marciano-Cabral and Cabral, 2003; Salah et al., 2009; Lamrabet et al., 2012; Zhang et al., 2014; Cueto et al., 2015; Lemmer et al., 2015; Paquet and Charette, 2016; Chan et al., 2018; Sedzicki et al., 2018; Garai et al., 2019), but the causes of this diversity are unknown. Distinct reactions to environmental stress can be a reason for such heterogeneity, as shown for aquatic microorganisms (Silva et al., 2016). However, direct links between physiological state, stress factors, and the bacterial ultrastructure have not been demonstrated. Identification of such links could delineate indicators of changes or circumstances critical for bacterial survival, to predict formation of persisters, to estimate sensitivities of populations, or to develop preventive strategies against bacterial infections. Hence, the investigation of different ultrastructural types and their frequencies may allow to predict prevailing environmental conditions, especially in the background of analyses of intracellular pathogens, or analyses of bacterial populations *in vivo* or free-living isolates.

The ability of *Salmonella enterica* serovar Typhimurium (STM) to survive harsh conditions within and outside of mammalian hosts makes it a good model organism to reveal the basis of bacterial heterogeneity. STM is a foodborne pathogen, capable to pass the low pH of the stomach. STM is exposed to various host defense mechanisms and competing microbes in the host gastrointestinal tract. STM can invade epithelial cells, survive and proliferate, and can abuse phagocytes for intracellular replication and systemic spread. Within host cells, STM is residing in a specific vacuole (*Salmonella*-containing vacuole, SCV) and drives the formation of a tubular membrane network (*Salmonella*-induced filaments, SIF), supporting its intracellular survival and progression (Liss and Hensel, 2015b; Gao et al., 2018; Göser et al., 2019). Moreover, STM is capable to survive and replicate within the host cell cytoplasm after escaping the SCV (Malik-Kale et al., 2011).

Intracellular survival requires fast stress response and cellular reprogramming for protection and repair when facing strong bactericidal host defenses such as reactive oxygen species (ROS) (Shen and Fang, 2012; Kröger et al., 2013; Liss et al., 2017; Noster et al., 2019b). The oxidative stress response in bacteria is one of the most crucial response to maintain cell viability. The activators of the superoxide regulon SoxRS and the hydrogen peroxide sensor OxyR induce the oxidative stress response (Vega et al., 2012; Wang et al., 2017). Other important proteins in the cytoplasm are the two manganese- and iron-dependent superoxide dismutases SodA and SodB, which are regulated by the SoxRS regulon (Fujikawa et al., 2012). The induction of the SoxRS regulon and resulting increased expression of superoxide dismutases ensures ROS neutralization and repair of cellular structures damaged by ROS.

In this study, we shed light on the ultrastructural consequences of environmental changes and exposure to stressors. We demonstrate that ultrastructural heterogeneity depends on the environment, and can be induced experimentally. The approach presented here enables to link ultrastructural heterogeneity with the physiological status of individual bacteria and environmental cues. For that, we combined classical microbiological assays with qualitative and quantitative TEM to study effects of induced oxidative stress and bactericidal conditions in STM wild type (WT) and a Δ*sodAB* strain hypersensitive to ROS (Noster et al., 2019b). Furthermore, we developed a strategy for fast correlative light and electron microscopy (CLEM) using high-voltage TEM of thick serial sections, and a fluorescent reporter for measuring bacterial biosynthetic activity. These results validate that different ultrastructural types represent viable bacteria. The combination of ultrastructural studies at the single cell level with fluorescent reporters is the next step towards an understanding of bacteria as individual organisms.

## Results

### Ultrastructural heterogeneity of *Salmonella enterica* cells in culture

Bacterial cells rapidly respond to changing environments in order to adapt and to survive. We reasoned that response to different environments, stressors, or antimicrobial agents might result in cells differing in their ultrastructure. We asked if ultrastructural features reflect different physiological states of bacteria.

**Figure 1:**
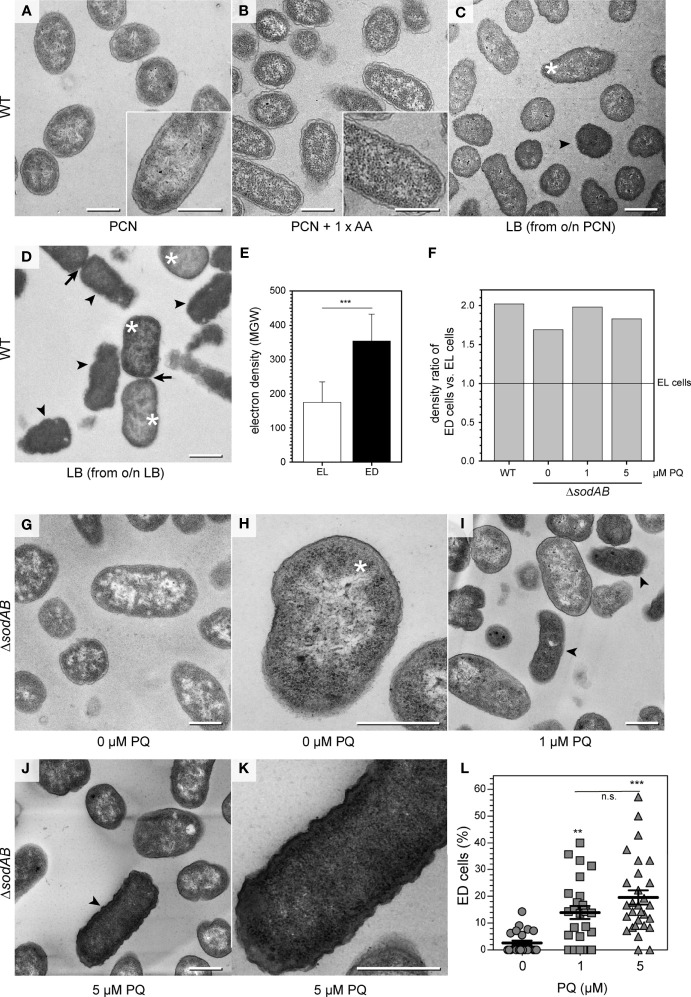
Environmental stress affects STM ultrastructure. **(A–D)** Energy-filtered transmission electron microscopy (EF-TEM) micrographs (120 keV) showing electron-lucent STM WT after growth in PCN medium **(A)**, PCN medium supplemented with AA **(B)**, and electron-lucent (asterisks) and electron-dense (arrowheads) STM WT subcultured in LB broth for 3.5 h **(C, D)**. Arrows indicate cells undergoing division. **(E)** Comparison of STM WT densities in LB medium shown as mean ± SD of different values between bacterial cytoplasm mean grey values (MGV) and background MGV (see **Figures S1A, B**, pooled data). **(F)** Comparison of density ratios of electron-dense **(**arrowheads in **C, D, I, J)** to electron-lucent cells **(**asterisks in **C, D, H)** of STM WT or STM Δ*sodAB* cultured in LB broth without or after PQ treatment. Numbers of cells quantified: 30, 22, 32, and 33, for STM WT, Δ*sodAB* 0 µM PQ, Δ*sodAB* 1 µM PQ, and Δ*sodAB* 5 µM PQ, respectively. **(G-K)** TEM micrographs showing STM Δ*sodAB* without **(G, H)**, or after treatment with PQ at 1 µM **(I)**, or 5 µM **(J, K)**. **(L)** Quantification of frequency of ED type cells. Each data point was generated from cell counting in TEM micrographs obtained at 120 keV, combining data from two biological replicates. Numbers of cells quantified: 318, 417, and 309, for 0 µM, 1 µM, and 5 µM PQ, respectively. Scale bars, 500 nm. Statistical analysis was accomplished by Student’s *t*-test **(E)**, and one-way ANOVA followed by Tukey’s Test between all pairs **(L)**. Significance levels are indicated as **, p < 0.01; ***, p < 0.001; n.s., not significant.

First, we examined STM WT ultrastructure using conventional TEM to find out if ultrastructural features depend on growth conditions, and which parameters are relevant (**Figures 1A–E**, **Figure S1**). STM WT was grown in fully defined synthetic PCN medium [Phosphate, Carbon, Nitrogen, (Neidhardt et al., 1974)] at pH 7.4 for 3.5 h for culture at a reduced growth rate of 0.89 h^-1^, compared to rich lysogeny broth (LB) with a growth rate of 2.13 h^-1^ (**Table 1**). Addition of a mixture of 20 amino acids (1 x AA) to PCN, pH 7.4 medium increased the growth rate of STM WT to 1.6 h^-1^ (**Table 1**), which is still lower than the growth rate in LB. STM WT grown as 3.5 h subcultures in PCN media consisted of very homogenous population of cells, independently of pH or AA supplementation (**Figures 1A, B**, **Figures S1H, J, L**). STM WT grown in LB was heterogeneous with profiles of clearly visible nanostructures and compartments, resembling PCN grown STM WT or with indistinguishable periplasm and nanostructures. Additional post-contrasting of ultra-sections with uranyl acetate and lead citrate improved visibility of compartments at low magnification only for one type, named as electron translucent (EL, asterisk in **Figures 1C, D**) since cytoplasmic region occurred as background for nanostructures. In contrast, type with unidentifiable nanostructures had high electron density after post-contrasting, referred as ‘electron- dense’ type (ED, arrowhead in **Figures 1C, D**) since it showed high capability of lead binding, what is evident for proteins. Furthermore, averaged electron density of cytoplasmic region measured as the mean gray value (MGV) was of 176 ± 60 MGV and 354 ± 78 MGV for EL and ED cells of standard growth conditions (3.5 h subculture in LB of o/n LB culture), respectively (**Figure 1E**). Both cell types were visible during cell division as an evidence of their viability (**Figure 1D**, arrows). Remarkably, STM WT 3.5 h PCN subcultures of o/n LB were homogenous EL populations despite o/n LB cultures were heterogeneous, and 3.5 h LB subcultures of o/n PCN did not show high number of ED, in contrast to standard LB growth condition (**Figures 1A, C, D**). The increased growth rate by AA supplementation did not increase ED frequency, indicating that factors other than rapid proliferation cause ultrastructural heterogeneity. Taken together this suggests that EL type is default and ED type occurs in response i.e. to environmental stress.

**Table 1:** Growth rates (in h^-1^) of STM WT and Δs*odAB* in various culture media*.

	WT		Δs*odAB*	
Medium**	mean	± SD	mean	± SD
LB	2.13	0.05	0.83	0.01
PCN 0.4, pH 5.8	0.73	0.07	0.18	0.31
PCN 0.4, pH 5.8 + AA	1.18	0.11	0.66	0.31
PCN 25, pH 7.4	0.89	0.04	0.41	0.06
PCN 25, pH 7.4 + AA	1.60	0.35	0.88	0.24

* cultures were grown in baffled flasks in a water bath set to 37°C with agitation at 160 rpm. Means and standard deviations (SD) of three biological replicates are shown.

** LB, lysogeny broth; PCN minimal media with 0.4 mM P_i_ at pH 5.8, or 25 mM P_i_ at pH7.4. Supplementation with 20 amino acid mix is indicated as + AA.

We further examined bacterial ultrastructural features for signs of cell death. In stationary LB cultures (**Figures S1E, G**), we found some dying cells, with partially or completely loss of inner membranes, signs of molecular condensation (dark spots, arrows in **Figure S1E**), and/or lysis (the arrowhead in **Figure S1G**). These profiles were also frequent in STM WT of o/n PCN cultures and independently of medium pH or AA supplementations (**Figures S1I, K, M**). In 3.5 h subcultures of corresponding media, profiles indicative of dying cells were only sporadically found (**Figures S1H, J, L**). We also noticed but sporadically and only in LB cultures, profiles with mixed density of cytoplasm. Such profiles had clear center surrounded by a denser ‘halo’. Therefore, we further applied high resolution TEM analysis to better define EL type with the uniform cytoplasm. The EL type had only denser periplasm as a compartment, measured as the distance between the outer and inner membranes of 20 ± 5 nm (mean ± SD). The center of cells, outlined by the inner membrane, was less electron dense with a difference of 135 ± 16 in MGV to the background. This region contained cytoplasm with protein complexes like ribosomes, visible as denser particles, which were distributed homogeneously. Electron-lucent and ribosome-free regions consisted of up to 16% of total cytoplasmic area and occupied areas up to 28 nm^2^ of clearly visible nucleoids.

Hence, depending on growth conditions, bacterial populations consist of either ultrastructural similar cells, or cells divergent in cytoplasmic electron densities.

### Controlled induction of STM ultrastructural heterogeneity

In order to find a correlation between ultrastructural types and environmental stress factors, we deployed the STM Δ*sodAB* strain deficient in both cytoplasmic superoxide dismutases SodA and SodB. STM Δ*sodAB* is especially sensitive to oxidative stress and turned out highly susceptible, being able to grow in LB, but very poorly in PCN (Noster et al., 2019b) (**Table 1**). Next, we induced oxidative stress by adding methyl viologen (‘paraquat’, PQ), a redox-active compound producing superoxide. We examined STM WT and Δ*sodAB* from 3.5 h LB subcultures, using propidium iodide (PI), a non-cell permeable DNA stain entering cells with damaged membranes. Colonies of STM Δ*sodAB* frequently contained high numbers of PI-positive cells without PQ treatment. PI staining was applied as a quality control prior to TEM, and experiments were continued only when the majority of cells without treatment were PI-negative (**Figure S2**). TEM showed STM Δ*sodAB* were ED and EL cells with ultrastructural features of STM WT (**Figures 1F, G**, **Figures S1F, G**), however the EL type was dominant (**Figures 1G, H**, asterisk). Treatment for 1 h at low concentrations of 1 or 5 µM PQ did not affect STM Δ*sodAB* growth on LB agar plates, however, TEM revealed that these treatments increased frequency of ED cells (**Figures 1I–K**, arrowheads) in a dose-dependent manner (**Figure 1L**), supporting the PQ treatment as causative. These results suggest that ED cells represent a type responding to cellular stress, e.g. induced by ROS.

**Figure 2:**
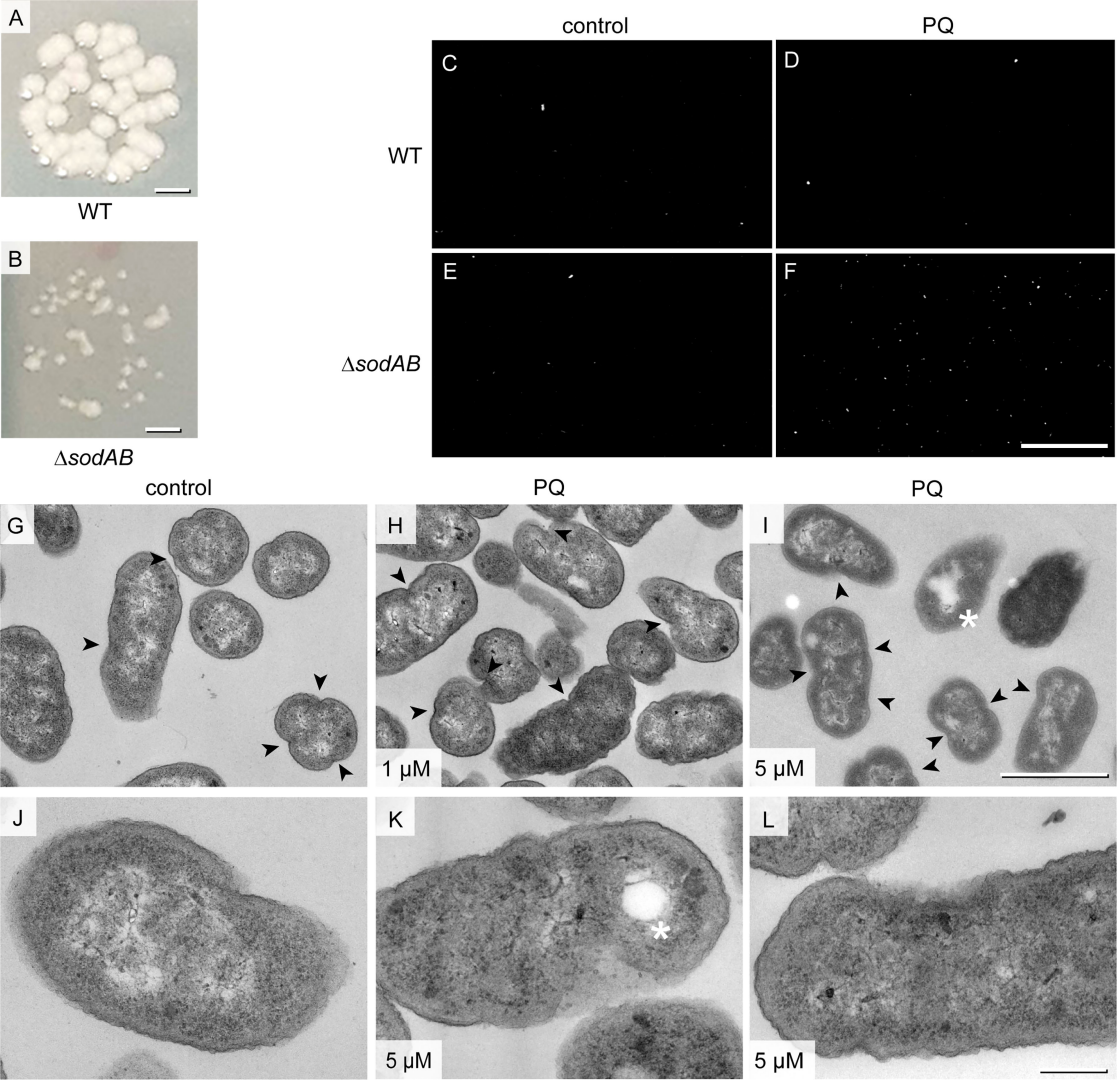
Growth defects and membrane abnormalities of STM Δ*sodAB*. **(A, B)** Colony growth of STM WT and STM Δ*sodAB* on LB agar plates. **(C–F)** Representative epifluorescence micrographs of PI staining of STM WT **(C, D)** or STM Δ*sodAB* **(E, F)**, without addition of PQ **(C, E** control**)**, or after treatment with 5 µM PQ **(D, F)**. Total numbers of cells per view of view were store by brightfield microcopy, and PI-positive cells were determined in same fields of view by epifluorescence microscopy. Number of cells (total/PI-positive) were: 557/10 **(C)**, 1504/9 **(D)**, 545/13 **(E)** and 379/161 **(F)**. Further conditions of PI staining are shown in **Figure S2**. **(G–L)** TEM of Δs*odAB* without treatment **(G, J,** controls**)**, or with addition of PQ to 1 µM **(H)**, or 5 µM **(I, K, L)**. Cells were fixed at 0 h post PQ treatment for preparation for TEM analysis and imaged at 120 keV EF-TEM. STM Δ*sodAB* treated with 5 µM PQ shows membrane rupture (arrowhead) and lysis spots (asterisks). Scale bars, 1 mm **(A, B)**, 100 µm **(C-F)**, 1μm **(G-I)**, 200 nm **(J-L)**.

To further scrutinize the link between ultrastructure and cellular stress, we analyzed growth phenotypes of STM Δ*sodAB*. STM Δ*sodAB* showed abnormal colony growth on agar plates, forming evidently smaller colonies (**Figures 2A, B**). Also the PI test showed that STM Δ*sodAB* had an increased proportion of PI-positive cells after application of 5 µM PQ, while STM WT was not affected (**Figures 2C–F**). High-resolution ultrastructural analysis of STM Δ*sodAB* revealed high number of cells with membrane invaginations. These were often asymmetrical, and single or multiple events occurred, which were differently located also including cell poles, therefore representing abnormal cell envelopes (**Figures 2G–J**). These features were also present in Δ*sodAB* without treatment (46%), but increased after treatment with 1 µM PQ (61%) or 5 µM PQ (59%), suggesting oxidative stress as cause of these effects (**Figures 2G–J**, arrowheads). Furthermore, in line with PI test, colonies with higher PI number after 5 µM PQ treatment harbored cells with damaged inner and outer membranes, manifested by a loss of integrity and leakage of cytoplasmic content (**Figures 2K, L**). Foci of lysis were also present after PQ treatment (asterisks in **Figures 2I, K**). Hence, PQ treatment enhanced cellular stress of STM Δ*sodAB*.

### Induction of ultrastructural heterogeneity of STM WT in PCN medium

As pathogenic bacterium, STM possesses multiple stress response systems that activate repair mechanisms to protect from oxidative stress, thus increasing the chance to survive PQ treatment (Ezraty et al., 2017). Therefore, we defined a toxic PQ concentration, which reduced the number of viable STM, and analyzed the ultrastructure of STM WT after PQ treatment in various growth conditions (**Figure S2**, **Figure S3**). PCN medium at pH 5.8 was used to mimic the acidic phagosomal lumen of macrophages, where superoxide is protonated, capable to pass bacterial membranes but less spontaneously dismutases into hydrogen peroxide (Slauch, 2011; Burton et al., 2014). STM WT grown o/n in PCN medium at pH 7.4 was inoculated in the same medium and grown further for 3.5 h. PQ treatments were always performed in PCN medium with reduced concentration of inorganic phosphate (P_i_) of 0.4 mM, since P_i_ could compete with PQ during transport through bacterial membranes (Kitzler and Fridovich, 1986). Shift to medium of acidic pH had little impact on viability, as compared to shift to PNC pH 7.4, 96% survival of STM shifted from PCN pH 7.4 to PCN pH 5.8 was determined. After treatment with 100 µM PQ, significant drop of survival to 20% or less of controls was observed at both pH values. Treatments with higher PQ concentrations such as 500 µM or 1 mM, further reduced survival, however, remained above 10% of controls (**Figure S3W**).

**Figure 3:**
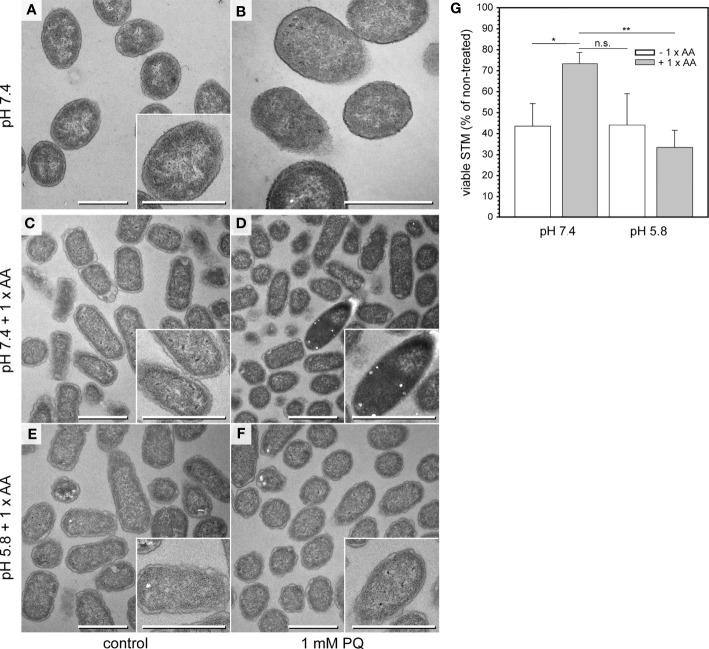
Emergence of ED cells after PQ treatment of STM WT in PCN pH 7.4 medium supplemented with AA. **(A, B)** TEM micrographs of STM WT cultured in PCN medium, pH 7.4 for 3.5 h and shifted to fresh PCN medium, pH 7.4 for incubation without **(**control **A)** or with 1 mM PQ **(B)**. **(C–F)** TEM micrographs of STM WT cultured in PCN medium pH 7.4 **(C, D)**, or pH 5.8 **(E, F)** supplemented with AA for 3.5 h, and shifted to the same fresh PCN medium for incubation without **(**control **C, E)**, or with 1 mM PQ **(D, F)**. Scale bars, 1 µm. **(G)** CFU counts obtained for STM WT with addition of 1 mM PQ. STM was subcultured for 3.5 h in PCN with or without AA supplementation, at pH 7.4 or pH 5.8. Means and standard deviations of three biological replicates are shown. Statistical analysis was accomplished by Student’s *t*-test and significance levels are indicated as follows: *, p < 0.05; **, p < 0.01; n.s., not significant.

TEM analysis of PQ-treated samples and controls revealed that in all conditions, STM cells were uniform in ultrastructural appearance when cultured in PCN at pH 7.4 or pH 5.8 without AA supplementation (**Figures 3A, B** and **Figures S3A–F**). After PQ treatment, the cytoplasm was denser but structures like inner membranes, ribosomes or DNA were easily distinguishable (**Figures S3G–L**). We did not find ED cells and only few cells with profiles of ultrastructural abnormalities. It is possible that in PCN medium with limited nutrients, bacteria were not capable to switch to an emergency mode after PQ treatment, what would explain absence of ED cells and poor growth on agar plates. To test this hypothesis, we performed the same experiments in PCN medium supplemented with AA, referred to as PCN + AA (**Figures 3C–F**). STM survival after treatment with 1 mM PQ was only higher when cultured in PCN, pH 7.4 + AA, in contrast to STM grown without AA, or in PCN, pH 5.8 (**Figure 3G**). This was in line with presence or absence of STM with ED type. ED cells emerged only in PCN, pH 7.4 + AA (**Figure 3D**). Treatment with lower PQ concentrations did not affect STM ultrastructure in PCN + AA (**Figures S3M–O**). For comparison, we also investigated the impact of other stress conditions on STM ultrastructure (**Figures S3P–V**). ED type was not observed after osmotic shock or heat shock. It occurred after acid shock (shift to pH 3.0) of STM subcultured in PCN, pH 7.4 + AA, but not after subculture in PCN, pH 5.8. We observed other ultrastructural features, which were specific to respective shock conditions and never observed in bacteria of control cultures. We compared presence of bacteria with shrinkage and/or lysis features since such profiles were observed during normal growth conditions (**Figure S3Y**). Cells with shrinkage and/or signs of lysis dominated the population after hyper-osmotic stress in presence of 600 mM NaCl (**Figure S3U**). Obvious increase of STM with signs of shrinkage and/or lysis was also observed after pH shock (pH 3.0) and was more pronounced when cells were subcultured at neutral pH. Simultaneous treatment with 1 mM PQ during pH shock resulted in comparable frequencies, suggesting only minor or no impact of PQ on causing shrinkage or lysis. This was in line with a low frequency of signs of shrinkage and/or lysis (< 10%) after PQ treatment in all other tested conditions (**Figure S3Y**). To conclude, occurrence of ED type is induced by environmental stress and requires presence of AA in culture medium.

### Ultrastructural heterogeneity of intracellular STM

**Figure 4:**
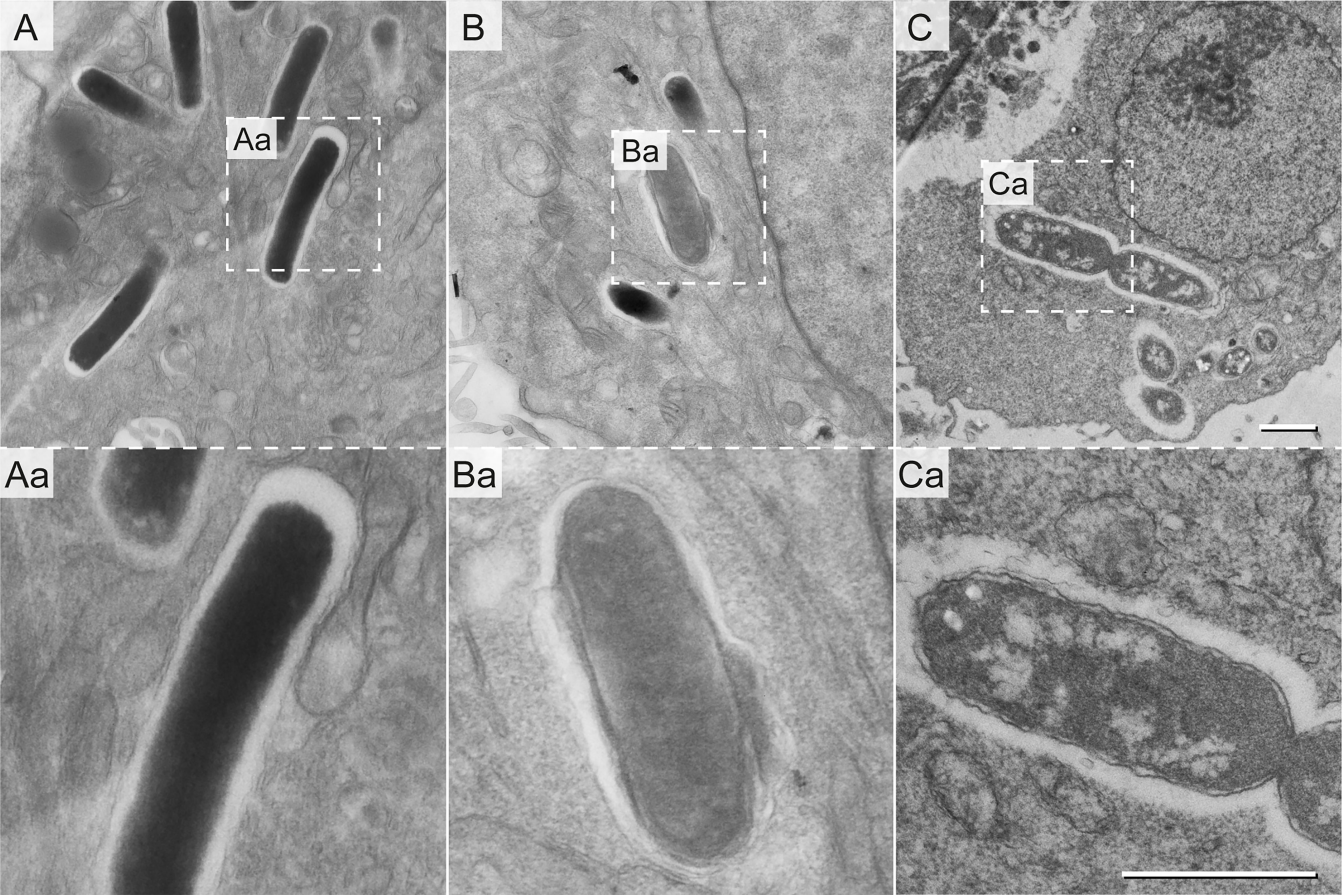
Ultrastructural heterogeneity of intracellular STM WT. STM WT was subcultured in LB for 3.5 h and used to infect HeLa cells. Infected cells were fixed 16 h p.i. and analyzed by EF-TEM at 120 keV. Micrographs show representative electron-dense **(A)** and electron-lucent STM WT **(B, C)**. Dividing STM WT cells are shown in **(C)**. Dashed boxes indicate areas enlarged in **(Aa, Ba,** and **Ca)**. STM cells shown are representative for morphology observed in at least three biological replicates. Scale bars, 1 µm.

Within eukaryotic host cells, STM encounters host cell defense mechanisms, as well as harsh phagosomal environments and nutritional limitations, yet STM is able to survive and to proliferate (Liss et al., 2017; Noster et al., 2019a). To correlate the ultrastructural features to intracellular phenotypes, we examined the ultrastructure of STM in HeLa cells at 8 h or 16 h post infection (p.i.). At both time points, host cells were either intact, with or without intracellular STM, or dying and ruptured as result of bacterial proliferation. Within healthy host cells, we found EL STM WT, as well as mixed populations with EL and ED cells similarly to STM in LB medium (**Figure 4**). Both types were located within SCVs and showed signs of cell division. These data confirmed that EL and ED types are ultrastructural morphotypes of STM occurring during infection.

### Biosynthetic activity of intracellular STM

To interrogate the biosynthetic capacity of distinct morphotypes of STM as proxy of metabolic activity, we used an episomal encoded dual-fluorescence reporter (**Figure S4A**) (Jennewein et al., 2015). Bacteria harboring the reporter constitutively express *gfp*. To report biosynthetic capacity, we monitored *dsred* expression regulated by the anhydrotetracycline (AHT)-inducible *tetA* promoter (Schulte et al., 2019). We considered cells as biosynthetic active when DsRed was detected after AHT induction.

**Figure 5:**
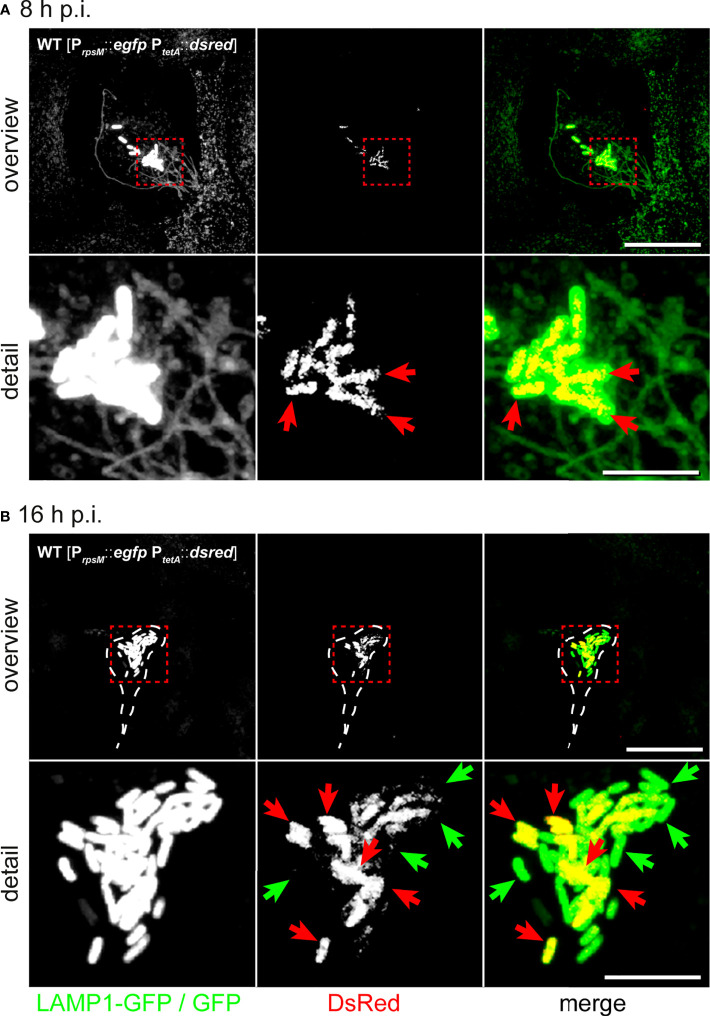
Hyper-replicating intracellular STM WT forms subpopulations of metabolically active and inactive bacteria. Intracellular STM WT harboring dual-fluorescence reporter in HeLa LAMP1-GFP cells were visualized 8 h **(A)** or 16 h p.i **(B)**. *Gfp* was constitutively expressed in STM WT, while *dsred* expression was induced by addition of AHT at 2 h prior to imaging. **(A)** DsRed is visible in all intracellular STM WT associated with SIF formation 8 h p.i. (yellow cells in merge, red arrows). **(B)** At 16 h p.i., hyper-replicating intracellular STM WT either lack DsRed (inactive cell indicated by green arrows) or are DsRed-positive (active cells indicated by red arrows). Micrographs are representative of phenotypes observed in at least three biological replicates. Scale bars, 20 µm (overview), 5 µm (detail).

First, we tested reporter functionality by live cell fluorescence microscopy (**Figure S4B**). STM WT harboring the reporter was used for infection of HeLa LAMP1-GFP cells. AHT was added 4 h p.i. with or without addition of chloramphenicol (Cm) to inhibit protein biosynthesis. DsRed fluorescence increased after AHT induction. Without AHT induction, or with AHT induction in presence of Cm, no DsRed-positive cells were detected (**Figure S4B**). Then, we investigated infected HeLa LAMP1-GFP cells 8 h or 16 h p.i. (**Figure 5**). The intracellular population was heterogeneous based on protein synthesis and divergent when compared between infected host cells consisting of either only metabolically active STM, or mixtures of metabolically active and inactive STM.

Previous TEM analysis showed that host cell viability decreased when containing high numbers of intracellular bacteria. Using LAMP1-GFP as marker, we assessed *Salmonella*-induced endosomal remodeling (**Figure 5A**). We also observed highly reduced LAMP1-GFP signals (**Figure 5B**), possibly due to activation of host cell death, or due to their rupture by escaping STM into the host cell cytoplasm. Presence of individual metabolically inactive STM within a population of metabolically active STM raised the question if inactive STM are viable. Bacteria may form persisters with ceased growth, highly reduced metabolism, and the ability to return to normal growth after release from stressful conditions. To further scrutinize the bacterial conditions in host cells, we applied correlative light and electron microscopy (CLEM).

### Correlation of biosynthetic activity and ultrastructural morphotypes

**Figure 6:**
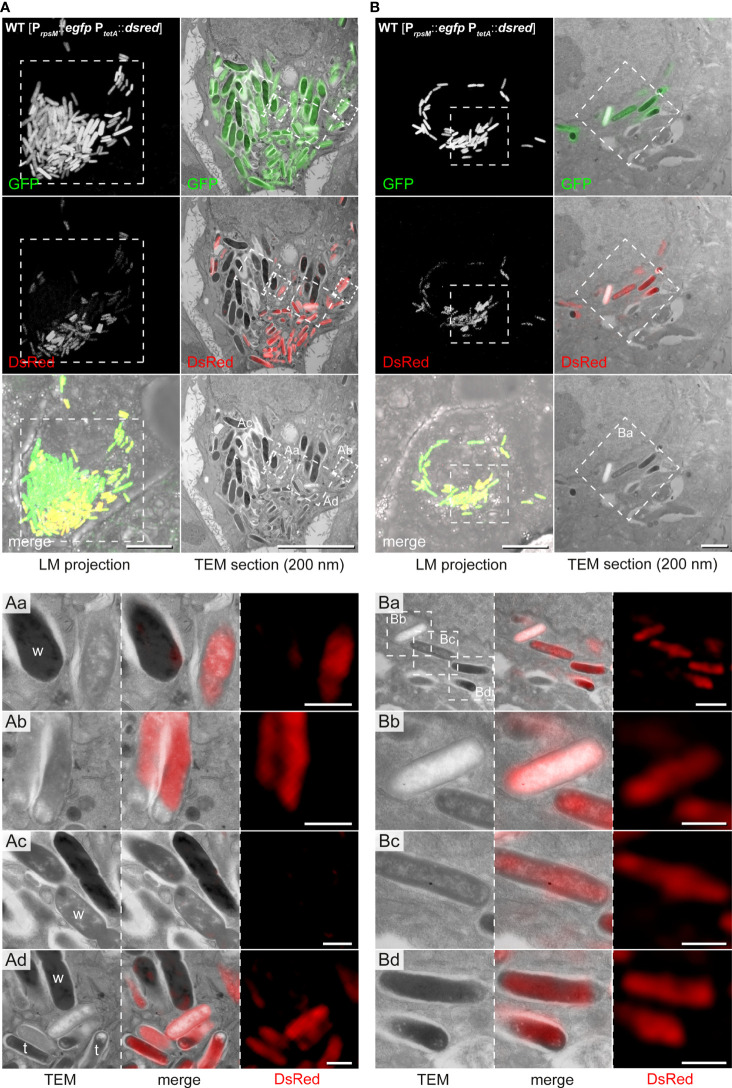
CLEM reveals ultrastructural and metabolic heterogeneity of hyper-replicating intracellular STM WT in HeLa cells. HeLa cells seeded on gridded cover slips were infected with STM WT harboring the dual-fluorescence reporter and visualized 16 h p.i. *Gfp* was constitutively expressed in STM WT while *dsred* expression was induced by AHT 2 h prior to imaging by confocal FM. Subsequently, cells were processed for TEM. **(A)** Representative HeLa cell with hyper-replicating intracellular STM WT, either DsRed-positive (active cells) or DsRed-negative (inactive). **(B)** Representative HeLa cell with replicating STM WT, which are DsRed-positive. For CLEM, EF-TEM micrographs of 200 nm thick sections of infected HeLa cells obtained at 120 keV were correlated with GFP or DsRed fluorescence signals of confocal sections after deconvolution. a-d) High-resolution CLEM of ROIs (white boxes) harboring intracellular STM WT of diverse electron density (TEM) and activity levels (DsRed, FM). Note ED and EL STM during division **(Ac)**, which are DsRed-negative. ‘Wide’ (w) and ‘thin’ (t) STM are marked. Micrographs are representative of phenotypes observed in at least three biological replicates. Scale bars, 10 µm (A, all overviews and B, LM overviews), 1 µm **(B,** TEM overviews, **Aa-Ad** and **Ba)**, 500 nm **(Bb–d)**.

We modified our CLEM approach (Liss and Hensel, 2015a) to accelerate data collection and further applied deconvolution of FM data (**Figure S5**). We observed intracellular STM populations consisting of ED and EL types (72.7% ED type, 27.2% EL type), which were also visualized during cell division (**Figures 6A, Ac, B**). Highly electron-lucent single bacteria showing clearly visible outer membrane, outlined periplasm, and produced DsRed at high level (**Figures 6Ad, Ba, b**). However, there was no strict correlation of biosynthetic active, less active, or inactive STM with any of electron density-based morphotypes. We found both, ED and EL types strongly (34.4% of ED and 41.7% of EL) or slightly (18.8% of ED and 16.7% of EL) marked for DsRed expression. In addition, 46.9% and 41.7% of ED and EL cells, respectively, were metabolically inactive. In ROIs with high numbers of STM, we detected clear differences in bacterial size, with areas of 1.76 ± 0.25 µm^2^ (wide) or 0.96 ± 0.18 µm^2^ (thin), which were only partially correlated with the electron density type (**Figure 4A**). Interestingly, size-based classes were rather grouped in the host cell with ‘wide’ STM located more centrally and ‘thin’ STM located on the cell peripheries (**Figure 6A**). Thin STM were highly metabolically active, while ED wide cells had no or minor expression of DsRed, localized to small patches. All together, these data provide further evidence for the existence of different ultrastructural classes.

**Figure 7:**
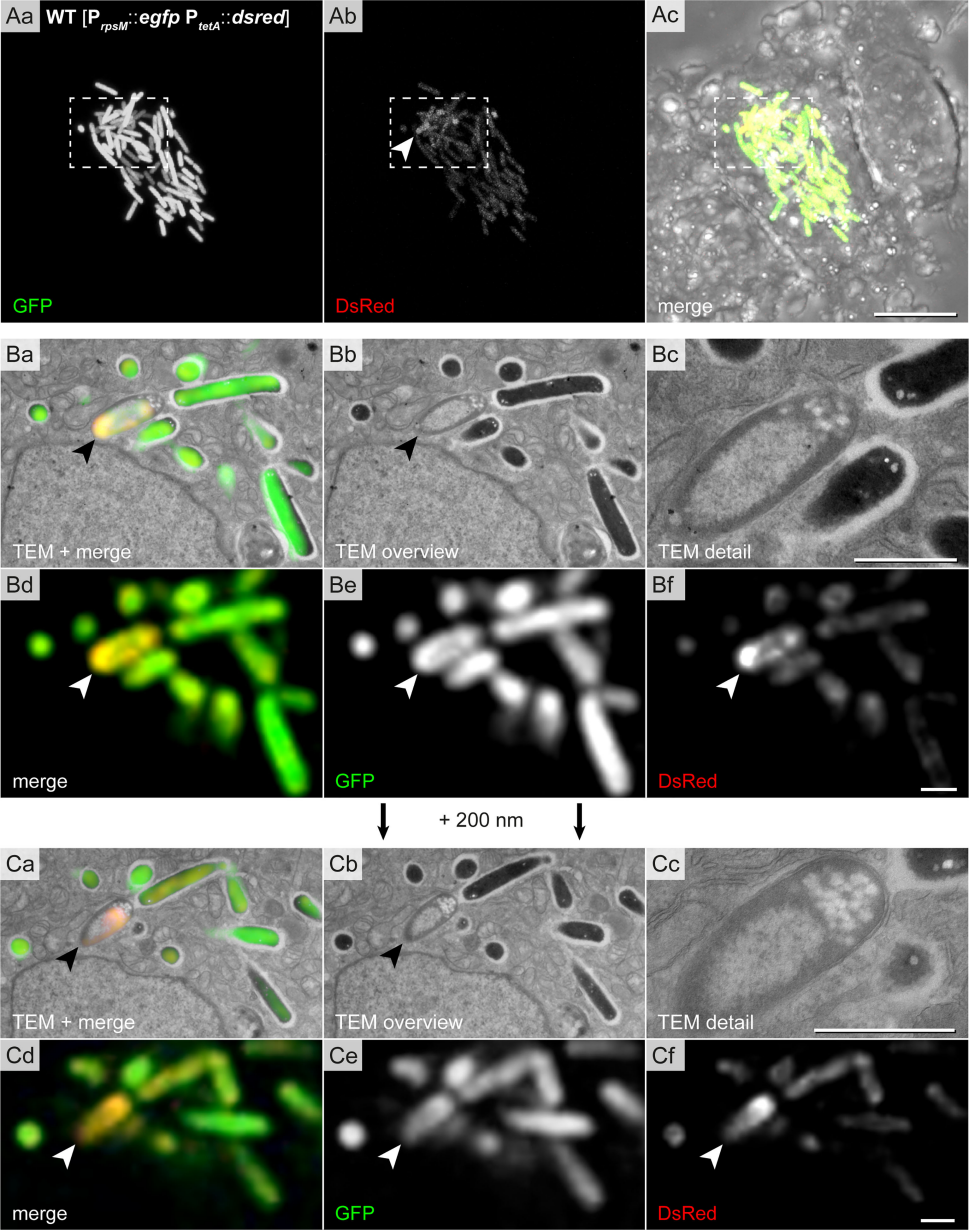
High-resolution CLEM precisely locates halo-shaped electron density to distribution of fluorescent proteins in single intracellular STM cells. Intracellular STM WT harboring the dual-fluorescence reporter in HeLa LAMP1-GFP cells were visualized. HeLa cell were seeded on gridded cover slips, infected with STM WT constitutively expressing *gfp*, while *dsred* expression was induced by addition AHT at 14 h p.i., prior to imaging by confocal FM at 16 h p.i. Subsequently, cells were processed for TEM. **(Aa–Ac)** Overview of HeLa cells with hyper-replicating intracellular STM WT (GFP, green) showing biosynthetic activity (DsRed, yellow in merge). Dashed boxes indicate the region further analyzed by CLEM. **(B, C)** CLEM of consecutive 200 nm thick sections of a region with STM WT of halo-shaped electron density (indicated by arrowheads). GFP and DsRed confocal fluorescence signals after deconvolution d-f are correlated with 120 keV EF-TEM micrographs a, b. GFP and DsRed are associated with halo-shaped electron densities. Micrographs are representative of phenotypes observed in at least three biological replicates. Scale bars, 10 µm **(A)**, 1 µm **(B, C)**.

Furthermore, we found STM cells with special features (**Figure 7**). These had clear condensations of structures in the cytoplasm with a dense layer surrounding loose materials in the center (halo-shaped condensation). Correlation of fluorescence signals with the ultrastructural profile showed that the halo-shape electron density contained GFP and DsRed, indicating high level of biosynthetic activity. At the poles, lucent blebs of regular size and shape were visible suggesting that they were not lysis spots. The bacterial inner membrane in proximity of lucent blebs was intact (**Figures 7B, C**).

Hence, the CLEM approach provided sufficient resolution to visualize distinct DsRed/GFP distributions inside bacteria in correlation with the ultrastructure.

### The ‘halo’ morphotype of bacterial cells

**Figure 8:**
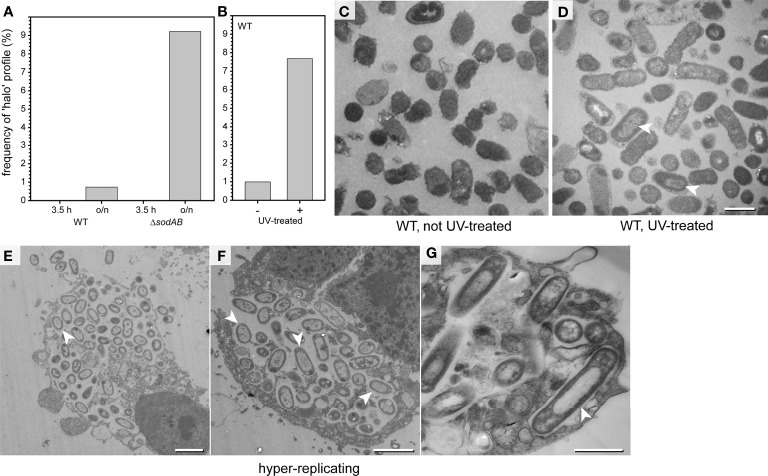
‘Halo’ type of STM WT dominates in critical environmental conditions. **(A)** STM was cultured in LB broth o/n, or further subcultured for 3.5 h in fresh medium prior to TEM preparation (TEM in **Figures S1D–G**). Comparison of relative numbers of STM WT and Δ*sodAB* cells with halo-shaped morphology. Number of cells quantified combining data from three biological replicates: 1,032, 819, 747, and 510, for WT 3.5 h, WT o/n, Δs*odAB* 3.5 h, and Δ*sodAB* o/n, respectively. **(B)** STM WT was irradiated 60 sec with UV light (305 nm) prior to TEM preparation. **(B)** Comparison of relative numbers of halo-shaped STM WT after UV irradiation **(**arrowheads in **D)** to non-UV-treated control **(C)**, i.e. the same LB culture without UV treatment. Numbers of quantified cells: 716 and 612, for UV-treated and non-treated groups, respectively. **(D)** TEM micrograph of UV-treated STM WT with halo-shaped profiles (indicated by arrowheads). **(E–G)** TEM micrographs of HeLa cells 16 h p.i. with STM WT. Infected HeLa cells containing hyper-replicating STM WT show disrupted cell membranes and signs of cell death. Nearly all intracellular STM WT have cytoplasmic densities distributed with halo-shaped distribution (arrowheads). Scale bars, 2.5 µm **(E, F)**, 1 µm **(C, D, G)**.

We noticed appearance of STM with halo-shaped condensations also in o/n LB cultures, allowing quantifications of this morphotype. Cells with halo-shaped condensations had a centrally located lucent region, occupying 39-55% of the whole cell area (48.5% ± 4.9). Quantification revealed that cultures in late-logarithmically growth did not contain any cells with halo-shaped condensations, in contrast to stationary cultures (o/n) (**Figure 8A**). Moreover, the Δ*sodAB* strain more frequently formed halo-shaped morphology, suggesting a link to stress (**Figure 8A**). In contrast, we did not detect halo-shaped condensations in any STM cell cultured in PCN medium, independently of growth phase, pH, AA supplementation, or even shock conditions.

We further induced the appearance of the halo-shaped type experimentally. UV irradiation at wavelengths of 290-320 nm (UVB), or 254 nm (UVC) is highly bactericidal (Coohill and Sagripanti, 2008). Therefore, STM WT cultured in LB medium were UV irradiated, resulting in complete lack of colony growth on agar plates. These cells were analyzed by TEM (**Figures 8B–D** and **Figures S6A-E**). The ultrastructural profiles of UV-treated STM were classified, revealing reduced numbers of EL type when compared to untreated cells **Figure S6C**). ED profiles were slightly more frequent, together with profiles showing signs of cellular death. UV treatment induced formation of the halo-shaped type to almost the frequency observed for STM Δ*sodAB* (**Figures 8A–D**, arrowhead in **D**). Hence, there is a correlation of the halo-shaped type frequency with occurrence of cell death induced by UV illumination.

We further analyzed infected host cells for presence of halo-shaped morphotype and found that this type dominated in intracellular populations in case of hyper-replication, when bacteria were leaving the host, or host cells were necrotic (**Figures 8E–G**, arrowheads). After growth in rich media like LB cultures, halo-shaped morphotype was observed in about 10% of cells, suggesting low exposure to cell-damaging agents. Within host cells, the impact on ultrastructural characteristics of STM was more dramatic. Taken together, these results demonstrate that the halo-shaped morphotype could serve as indicator of exposure of STM to critical environmental conditions.

## Discussion

In this work, we present evidence that several morphotypes of viable bacteria exist. Based on ultrastructural criteria, these morphotypes differ significantly in cytoplasmic electron density, which was well distinguishable already at low EM resolution (1,000-2,000-fold magnification, 50 keV), as well as in organization and visibility of defined (chromosome, ribosomes) and undefined structures (dense cytoplasmic areas). We identified EL and ED types of STM, representing viable bacteria active in protein biosynthesis and/or able to divide. The frequency of morphotypes in a bacterial population could be used as an indicator of environmental changes, what in turn enables new ways of EM data interpretation. Moreover, ultrastructural heterogeneity is not limited to *Salmonella enterica*, since published TEM micrographs of other bacteria demonstrate morphotypes similar to EL, ED, as well as halo-shaped type. These types seem neither to be restricted to Gram-negative, nor pathogenic bacteria. Examples of the morphotypes have been previously visualized for intracellular bacteria both in infections of cultured cells or animal models, and were not restricted to mammalian cells (Via et al., 1997; Szenasi et al., 1998; Inglis et al., 2000; Marciano-Cabral and Cabral, 2003; Salah et al., 2009; Lamrabet et al., 2012; Zhang et al., 2014; Cueto et al., 2015; Lemmer et al., 2015; Paquet and Charette, 2016; Chan et al., 2018; Sedzicki et al., 2018; Garai et al., 2019). However, the biological relevance of structural heterogeneity has been unclear. Because TEM image quality is highly affected by variations in procedures of sample preparation, such variation may be considered as causative to differences in morphotypes. Here, the experimental induction of ED morphotype and observations of different types, independently of fixation but depending on growth conditions, brought evidence that distinct morphotypes have a natural cause.

Heterogeneity in bacterial populations is a common observation and phenomena such as persister formation or bet hedging are consider as survival strategy under adverse conditions (Norman et al., 2015). Population heterogeneity has been described for *S. enterica*, for example with respect to host cell invasion (Ackermann et al., 2008), the intracellular fate in host cells (Birmingham et al., 2006; Knodler, 2015; Reuter et al., 2021), or persister formation (Fisher et al., 2017; Schulte et al., 2021). Here, we provide evidence that ultrastructural heterogeneity is associated with environmental changes. We further demonstrated that external AA supplementation is required for ED induction, and that oxidative stress-dependent formation of ED bacteria only takes place in nutrient-rich medium. In contrast, ED cells were hardly inducible by oxidative stress in minimal media without AA. Furthermore, induction of ED type is pH-sensitive with restriction to a phagosomal pH. Consistent with lower viability of STM in PCN medium, the lack of ED morphotype in PCN medium despite oxidative stress may reflect inability of cells to respond properly and to switch to emergency mode. This interpretation is also in a line with effects of *sodAB* defects, reflected by vulnerability of the cells and frequency of ED type. As expected, STM Δ*sodAB* is highly sensitive to oxidative stress, and TEM and PI staining demonstrated membrane damage at oxidative stress levels well tolerated by STM WT. ED frequency increased when oxidative stress was higher. Therefore, ED type can be considered as indicative of cells sensing and responding to stress. Interestingly, the expression of SsrAB regulon with genes encoding the SPI2-T3SS and effectors proteins responsible for endosomal remodeling by intracellular STM (Liss and Hensel, 2015b) is also activated by exposure to ROS (Kim et al., 2022). Future work may reveal if STM with ED type are also cells that express the SPI2-T3SS and actively translocate effector proteins to manipulate the host cell. The membrane damage observed suggests that STM Δ*sodAB* does not react sufficiently, has much delayed response for successful compensation, or membrane repair is not sufficient when damage is excessive and rapid. Previously, a morphotype reminiscent with ED has been described in *E. coli*, as representative of healthy cells (Kim et al., 2018; Wood et al., 2019).

At present we do not know the underlying mechanism, but one possible explanation for emergence of ED type can be macromolecular crowding as a result of stress response (McGuffee and Elcock, 2010; Vendeville et al., 2011). Previously, molecular crowding has been demonstrated in *E. coli* after osmotic up-shift, affecting diffusion of GFP. These changes were accompanied by dramatic cytoplasmic shrinkage, which was only sporadically observed at the cell poles by TEM in our experiments (Konopka et al., 2006; Mika et al., 2010).

Global and dramatic ultrastructural alterations of bacteria occurred very fast, because differences were visible by TEM when cells were fixed directly after stress treatment. On the other side, dead and dying bacterial cells, according to ultrastructural criteria like membrane injuries, lysis, and/or leakage of content are not frequent, even after bactericidal UV irradiation, or after exposure to shock conditions. This suggests that STM can be alive for a long time, yet unable to form colonies. We also visualized cells at high resolution to clearly discriminate between lysis and storage granules or other electron-lucent components not related with death (this study; Konopka et al., 2006; López-Heras et al., 2015), and between molecular condensation and protein-based organelles like carboxisomes (Penrod and Roth, 2006; López-Heras et al., 2015). Criteria indicative of dying cells were found in STM of both EL and ED types.

In addition, we have identified the very characteristic halo-shaped morphotype, which dominates bacterial populations exposed to extreme conditions. This morphotype occurred in stationary phase, was increased after UV irradiation, and in STM Δ*sodAB*, and was observed in intracellular bacteria, suggesting links to deadly stressors. CLEM analysis of halo-shaped morphotype cells suggests that the dense matter around the DNA-containing center is of proteinaceous nature, since GFP and DsRed were located within this area. A possible explanation for this phenomenon could be global DNA compaction. Organization and condensation of bacterial DNA is mediated by nucleoid-associated proteins (NAPs). The NAP Dps condenses the bacterial DNA, is the most abundant protein component of the nucleoid first described for *E. coli* (Almiron et al., 1992). For protection from severe damage, DNA can be condensed into a compact structure, as shown both *in vitro* and *in vivo* (Wolf et al., 1999; Kim et al., 2004). Since Dps is active during stationary phase and predominantly during exposure to stress conditions, we suggest that the halo-shaped type results as a consequence of DNA ‘biocrystal’ formation (Wolf et al., 1999). Since the halo-shaped type was found especially in intracellular bacteria, this underlines our hypothesis that *Salmonella* protects its DNA in harsh and stressful conditions. Nevertheless, further investigations of mutant strains lacking NAPs in combination with DNA stains e.g. by DAPI or Hoechst will shed more light on this phenomenon. A recent study analyzed a *E. coli* Δ*dps* mutant strain in combination with DAPI staining (Janissen et al., 2018). The study showed that DNA condensation mediated by Dps is decoupled from transcription and that access of proteins to the encased DNA is still provided by Dps.

Taken together, our study sheds light on ultrastructural heterogeneity of STM and reveals possible indicators for TEM analyses, which allows to broaden interpretation of EM data. The ED type could serve as indicator for oxidative stress, and the halo-shaped type as indicator for hazardous environmental conditions when the DNA needs to be shielded and protected. Future research in infection biology may use these indicators when other detailed analyses of single cell fate are difficult or impossible. For example, this may apply to bacterial populations *in vivo* in solid tissues, or to analyses of bacterial populations not compatible with reporter systems such as free-living microorganisms in their natural habitat.

## Materials and methods

### Bacterial strains and growth conditions

*Salmonella enterica* serovar Typhimurium strain NCTC 12023 (STM) was used as wild-type strain and isogenic strain MvP2400 (Δ*sodA*::FRT Δ*sodB*::FRT) has been described (Noster et al., 2019b). Bacteria were cultured in lysogeny broth (LB), or synthetic medium PCN supplemented with 0.4 mM (for paraquat treatment) or 25 mM PO_4_^-^ (Neidhardt et al., 1974; Popp et al., 2015), at pH of 5.8 or 7.4, at 37°C with aeration. Optionally, medium was supplemented with 20 amino acids, i.e. alanine (0.8 mM), arginine (5.2 mM), asparagine (0.4 mM), aspartate (0.4 mM), cysteine (0.1 mM), glutamic acid (0.6 mM), glutamine (0.6 mM), glycine (0.8 mM), histidine (0.2 mM), isoleucine (0.4 mM), leucine (0.8 mM), lysine (0.4 mM), methionine (0.2 mM), phenylalanine (0.4 mM), proline (0.4 mM), serine (10.0 mM), threonine (0.4 mM), tryptophan (0.1 mM), tyrosine (0.2 mM), valine (0.6 mM) (Neidhardt et al., 1974). When required, carbenicillin or chloramphenicol were added at 50 µg x ml^-1^ or 200 µg x ml^-1^, respectively. For live cell imaging of bacterial biosynthetic activity, the strains harbored plasmid pWRG658 (P*_rpsM_*::*gfpmut3A tetR* P*_tetA_*::*dsRed T3_*S4T) (Jennewein et al., 2015) for constitutive expression of *gfp* and inducible expression of *dsred*. Bacterial growth rate µ was determined by OD600 reads during culture in various liquid media and calculated as ln2/t_d_ (time of doubling).

### Induction by anhydrotetracycline

Non-antibiotic tetracycline analog anhydrotetracycline (AHT) (Fluka, Sigma-Aldrich) stock solutions of 200 µg x ml^-1^ in dimethylformamide (DMF) were stored in aliquots of -20°C in the dark. For induction of expression of the P*_tetA_*-controlled dual-fluorescence sensor, AHT was added directly to LB broth or cell culture medium to a concentration of 100 ng x ml^-1^ if indicated.

### Stress induction by methyl viologen, heat, hyper- or hypo-osmolarity

STM strains were cultured o/n as indicated, diluted 1:31 in fresh medium, subcultured for further 3.5 h, shifted to fresh PCN medium as indicated and exposed to methyl viologen (Sigma-Aldrich) for 1 h at RT without shaking, to 80°C (in PCN medium pH 7.4) or to hyper-osmolar (PCN medium pH 7.4 containing 600 mM NaCl) or hypo-osmolar conditions (pure H_2_O_dd_) for 2 h. Effect of methyl viologen was controlled by plating of bacteria onto LB plates. Subsequently, bacteria were processed for TEM as described above or below.

### UV inactivation of STM

O/n cultures of STM were grown in LB broth, normalized to an OD_600_ of 0.2 in PBS and transferred to a petri dish following irradiation with UV light (305 nm) for 60 sec. The effect of UV inactivation was always confirmed by plating of irradiated bacterial suspension onto LB plates. If indicated, inactivated bacteria were processed for EM as described below.

### Cell lines and cell culture

For infection experiments the non-polarized epithelial cell line HeLa (American Type Culture Collection, ATCC no. CCL-2) stably transfected with LAMP1-GFP was used. HeLa cells were cultured in Dulbecco’s modified Eagle’s medium (DMEM) containing 4.5 g x l^-1^ glucose, 4 mM stable glutamine and sodium pyruvate (Biochrom) and supplemented with 10% inactivated fetal calf serum (iFCS) (Sigma-Aldrich) at 37°C, 5% CO_2_ and 90% humidity.

### Host cell infection

For infection of HeLa LAMP1-GFP cells, *Salmonella* strains were grown o/n in LB broth, diluted 1:31 in fresh LB and subcultured for further 3.5 h to induce maximal invasiveness. Infection was performed with a multiplicity of infection (MOI) of 50 for 25 min at 37°C, 5% CO_2_ and 90% humidity. Subsequently, cells were washed thrice with PBS and incubated for 1 h with medium containing 100 mg x ml^-1^ gentamicin (Applichem) to kill all non-invaded bacteria. Afterwards, the medium was replaced by medium containing 10 mg x ml^-1^ gentamicin until the end of the experiment.

### Propidium iodide staining of STM

Propidium iodide (PI) (Sigma-Aldrich) was used to analyze cell envelope integrity. STM was cultured in LB, and incubated without or with PQ at indicated concentrations in PBS for 10 min in the dark. Subsequently, bacteria were washed twice by centrifugation (5,000 x g, 5 min) in PBS. For imaging, 5 µl of diluted bacteria were placed on a glass slide, covered with a cover slip and imaged by the Zeiss LSM (Zeiss), using EC Plan-Neofluar 40x/1.30 oil objective, 543 nm laser and filter 587 – 704.

### Live cell imaging and image deconvolution

For live cell imaging, DMEM was replaced by imaging medium consisting of Minimal Essential Medium (MEM) with Earle’s salts, without NaHCO_3_, without L-glutamine and without phenol red (Biochrom) supplemented with 30 mM HEPES (4-(2-hydroxyethyl)- 1-piperazineethanesulfonic acid) (Sigma-Aldrich) at pH 7.4. For imaging of fixed cells, cells were washed thrice with PBS and incubated for 15 min with PBS containing 3% *para*-formaldehyde (PFA) to ensure complete fixation of cells. Subsequently, cells were washed thrice with PBS and blocked with blocking solution containing 2% bovine serum albumin and 2% goat serum in PBS. Fluorescence imaging was performed using the confocal laser-scanning microscope (CLSM) Leica SP5. For setting adjustment, image acquisition and image processing the software LAS AF (Leica, Wetzlar, Germany) was used. Image acquisition was performed using objectives 10x (HC PL FL 10x, NA 0.3), 20x (HC PL APO CS 20x, NA 0.7), 40x (HCX PL APO CS 40x, NA 1.25–0.75) and 100x objective (HCX PL APO CS 100x, NA 1.4–0.7) (Leica, Wetzlar, Germany) and the polychromic mirror TD 488/543/633 for the three channels GFP, DsRed and DIC. For CLEM experiments, images were further deconvoluted using Huygens software (Scientific Volume Imaging B.V., Hilversum, The Netherlands) to better correlate the expression patterns of DsRed to the bacterial ultrastructure. Live cell imaging was performed using the Zeiss Cell Observer microscope with Yokogawa Spinning Disc Unit CSU-X1a, Evolve EMCCD camera (Photometrics, USA) and live cell periphery, equipped with an Alpha Plan-Apochromat 63x (NA 1.46) oil immersion objective (Zeiss, Oberkochen, Germany). Following filter combinations were used for image acquisition: GFP with BP 525/50, DsRed with LP 580 and processed by the ZEN 2012 (Zeiss, Oberkochen, Germany) software. Scale bars for all acquired images were added with Photoshop CS6 (Adobe).

### Sample preparation for TEM

STM cultured either in LB or PCN medium were fixed with 2.5% glutaraldehyde (GA) (Electron Microscopy Science) in 100 mM phosphate buffer (81.8 mM Na_2_HPO_4_ and 18.2 mM KH_2_PO_4_, pH 7.2) o/n at 4°C. Unreacted aldehydes were blocked with 100 mM glycine in buffer for 15 min. Osmification was performed with 1% osmium tetroxide (Electron Microscopy Science) in 100 mM phosphate buffer for 60 min on ice, followed by washing several times with phosphate buffer and ultrapure water (MilliQ). Subsequently, contrasting with 1% uranyl acetate (Electron Microscopy Science) in MilliQ for 30 min was performed followed by several washing steps. Afterwards, cells were dehydrated in a cold graded ethanol series finally rinsing once in anhydrous ethanol, and twice in anhydrous acetone at room temperature. Infiltration was performed in mixtures of acetone and EPON812 (Serva). After every incubation or washing step, bacteria were centrifuged (2,000 x g, 3 min), the supernatant was discarded, and bacteria resuspended for by the next preparation step.

### Sample preparation for CLEM

Two days prior to infection HeLa LAMP1-GFP cells (1 x 10^5^) were seeded onto a gridded coverslip in a petri dish (MatTek, Ashland, MA). 14 h p.i. 100 ng x ml^-1^ AHT was added to the cells for induction of reporter plasmid. 16 h p.i. cells were pre-fixed with pre-warmed 2.5% GA in 100 mM phosphate buffer for 15 min at 37°C. After washing the cells thrice with PBS, ROIs were registered and images acquired. Subsequently, further fixation was performed using 2.5% GA in 100 mM phosphate buffer o/n at 4°C. Quenching, osmification and contrasting was performed as described above. Then, the gridded coverslip was removed from the petri dish and was transferred to a glass dish. Afterwards, cells were dehydrated in a cold graded ethanol series, finally rinsing once in anhydrous ethanol and twice in anhydrous acetone at room temperature. Infiltration and flat-embedding were performed in mixes of acetone and EPON812 (Serva). During the removal of the gridded coverslip from the polymerized EPON the engraved coordinates were transferred to the EPON surface and allowed easy relocation by microscopy. ROIs were cut using a scalpel and transferred to an EPON block. Serial 200 nm sections were generated by an ultramicrotome (Leica EM UC7) and collected on formvar-coated copper EM slot grids.

### Transmission electron microscopy

High-resolution analysis including CLEM was performed using the Libra 120 TEM (Zeiss, Oberkochen, Germany) operating at 120 keV, equipped with an Omega energy filter and a 2,000×2,000-pixel digital camera (Troendle). In addition, TEM was performed using a Zeiss 902 system (Zeiss, Oberkochen, Germany) operating at 50 keV. Images were recorded using ImageSP software (TRS image SysProg, Moorenwies, Germany). If required, TEM micrographs were adjusted for brightness and contrast enhanced using ImageJ or Photoshop. For image analysis, ImageJ (http://rsbweb.nih.gov/ij/) was used. Stitching and overlay of CLSM and TEM images were done using Photoshop CS6 (Adobe).

## Data availability statement

The original contributions presented in the study are included in the article/**Supplementary Material**. Further inquiries can be directed to the corresponding author.

## Author contributions

MS, MH and KM designed the research, analyzed the data, and wrote the manuscript. MS and KM performed the research. All authors contributed to the article and approved the submitted version.

## Funding

This work was supported by the DFG by SFB944, subproject P15 to MH and KM, and subprojekt Z. MH was further supported by grant HE1964/23-1 within DFG priority program SPP 2225.

## Acknowledgments

The excellent technical assistance of Birgit Hemmis and Britta Brickwedde is kindly acknowledged.

## Conflict of interest

The authors declare that the research was conducted in the absence of any commercial or financial relationships that could be construed as a potential conflict of interest.

## Publisher’s note

All claims expressed in this article are solely those of the authors and do not necessarily represent those of their affiliated organizations, or those of the publisher, the editors and the reviewers. Any product that may be evaluated in this article, or claim that may be made by its manufacturer, is not guaranteed or endorsed by the publisher.

## Supplementary material

Supplementary Figure 1 (related to **Figure 1**): **(A-C)** Quantification of electron densities. STM WT or Δ*sodAB* strains were cultured for 3.5 h or o/n in various medias as indicated and ultrastructure was analysed by 120 keV EF-TEM. **(A)** Comparison of electron densities (difference between MGV of STM cytoplasm and MGV of background) of ED and EL STM WT in various regions of specimen. Averages of electron densities **(B)**, and averaged density ratio **(C)** (mean ± SD). **(D–G)** Electron micrographs were obtained for STM WT and Δ*sodAB* strains from o/n cultures or 3.5 h subcultures in LB broth at 50 keV. **(H-M)**, STM WT from o/n or 3.5 h subcultures in PCN, pH 7.4 with **(J, K)**, or without AA supplementation **(H, I)**, or in PCN, pH 5.8 **(L, M)**. Arrows in **E** indicate cells with mixed electron densities and halo-shaped distribution. Arrowheads in G indicate translucent spots in STM Δ*sodAB* cells of o/n culture not found in 3.5 h subculture. Scale bar, 1 µm. Data are combined from three biological replicates **(A–C)** and representative micrographs are shown **(D–M)**. Statistical analysis was accomplished by Student’s *t*-test, and significance levels are indicated by **, p < 0.01.

Supplementary Figure 2 (related to **Figure 2**): Paraquat treatment affects membrane integrity to similar extend in STM WT and Δ*sodAB* strains. **(A)** Relative numbers of PI-positive STM Δ*sodAB* without and with treatment with paraquat (PQ). Quantification of PI-positive cells in cultures after treatment with 5, 10 or 50 µM PQ was achieved in comparison to control cultures (no treatment). Number of cells quantified combined from three biological replicates: 3,876, 1,5825, 15,804, and 18,016, for 0 µM, 5 µM, 10 µM, and 50 µM PQ, respectively. **(B, C)** Effects of treatment of STM WT and Δ*sodAB* without or with 50 µM PQ on PI fluorescence intensities. The relative number of PI-positive cells and fluorescence intensities of PI. PI was added 12 h after PQ treatment. STM Δ*sodAB* shows high variability in number and degree (intensities) of PI-positive cells. Statistical significance between controls and PQ-treated cells was calculated by Student’s *t*-test and significance levels are indicated as *, p < 0.05; **, p < 0.01.

Supplementary Figure 3 (related to **Figure 3**): Treatment of STM WT with various stressors has impact on ultrastructure. **(A–F)** Electron micrograph of STM WT cultured in PCN, pH 7.4 for 3.5 h and shifted to fresh PCN, pH 7.4 **(A–C)**, or PCN, pH 5.8 **(D–F)** for incubation without PQ **(**control **A, D)**, with 100 µM PQ **(B, D-E)**, or 500 µM PQ **(C, F)**. **(G–L)** EF-TEM micrographs of STM WT without (control), after 100 µM or 500 µM PQ treatment, displayed as an electron density scale. Arrowheads point to cells with denser cytoplasm compared to controls. **(M–O)** EF-TEM micrographs of STM WT cultured in PCN, pH 7.4 supplemented with AA for 3.5 h and shifted to fresh PCN, pH 7.4 for incubation without **(**control **M)**, with 100 µM PQ **(N)**, or 500 µM PQ **(O)**. **(P–S)** EF-TEM micrographs of STM WT cultured in PCN, pH 7.4 or PCN, pH 5.8 supplemented with AA for 3.5 h and shifted to fresh PCN, pH 3.0 for incubation without **(P, R)**, or with 1 mM PQ **(Q, S)**. **(T–V)** EF-TEM micrographs of STM WT cultured in PCN, pH 7.4 for 3.5 h and shifted to fresh PCN, pH 7.4 for incubation at 80°C **(T)**, to PCN, pH 7.4 containing 600 mM NaCl **(U)**, or to pure H_2_O_dd_ **(V)**. **(W)** Aliquots of STM subcultured for 3.5 h in PCN, pH 7.4 or PCN, pH 5.8 without PQ treatment, or treatment with 100 µM, 500 µM or 1 mM PQ were plated onto agar plates, and CFU were determined. Bacterial survival is expressed as percentage of untreated culture. **(X, Y)** Relative numbers of STM WT with shrinkage **(**arrowhead in **D)** or features of lysis **(**arrowhead in **F)** in indicated culture conditions. Features of untreated control samples in % of total are shown in **(X)** and x-fold increase of features of stressor-treated samples compared to the respective untreated sample is shown in **(Y)**. In addition, values of shrinkage and lysis of stress-treated samples in % of the total population is indicated above. 100-300 cells were quantified cells for each condition, combined from three biological replicates. Scale bars, 1 µm **(A–V)**, 250 nm **(**detail in **C–F)**.

Supplementary Figure 4 (related to **Figure 5**-**7**): Functional characterization of dual-fluorescence reporter in STM WT. **(A)** The dual-fluorescence reporter harbors cassettes for constitutive *gfp* expression, and AHT-inducible expression of *dsred*. Metabolically active bacteria are expected to synthesize DsRed after addition of AHT, in contrast to metabolically inactive bacteria, or if protein biosynthesis is experimentally blocked by chloramphenicol (Cm) after induction. **(B)** Live cell imaging of intracellular STM WT with dual-fluorescence reporter (GFP) in HeLa cells 8 h p.i. Bacteria were DsRed positive (yellow in merge) only after addition of AHT. AHT was added 4 h p.i. Addition of Cm with AHT blocks DsRed synthesis. Scale bar, 5 µm.

Supplementary Figure 5 (related to and **Figure 6**, **7**): CLEM approach to analyze intracellular STM at high resolution. **(A)** To register coordinates for CLEM, HeLa cells were seeded at a gridded cover slip prior to infection with STM WT harboring the dual-fluorescence reporter. *Gfp* was constitutively expressed in STM WT, while *dsred* expression was induced by addition of AHT 2 h prior to imaging of selected ROIs by CLSM at 16 p.i. Subsequently, samples were fixed and prepared for TEM by flat-embedding in plastic resin. Coordinates from CLSM were well visible, allowing relocation of ROIs. Resin fragments containing ROIs were dissected and individually fixed to resin blocks for serial 200 nm ultra-sectioning. After relocation of HeLa cells containing STM WT in TEM modality, serial sections were acquired at 120 keV with energy filtering (Ω filter). This approach significantly reduced time needed for sample preparation to image whole cells with TEM, providing the opportunity to collect data of high quality at optimal resolutions for correlation or TEM analysis. **(B)** Examples of the CLEM workflow: visualization by CLSM of intracellular STM WT by virtue of GFP fluorescence, and evaluation of metabolic activity by virtue of DsRed intensity (Z-stack through STM population, maximum intensity projection is shown), registration of positions of infected HeLa cells using BF-LM, relocalization of HeLa cells in resin using imprinted CLEM coordinates, TEM imaging, correlation of single CLSM planes with corresponding single TEM images. **(C)** Comparison of fluorescence signals without and with deconvolution using Huygens software. After deconvolution, location of proteins GFP and DsRed was increased, and signal-to-noise ratio was improved. Scale bars, 20 µm **(B**, left panel**)**, 5 µm **(B,** right panel, **C)**, 2 µm **(C**, detail**)**.

Supplementary Figure 6 (related to **Figure 8**): UV treatment of STM WT increases ultrastructural heterogeneity. **(A, B)** Cultures of STM WT grown o/n in LB were irradiated 60 sec. with UV light (305 nm) prior preparation for TEM. As control, STM WT of the same culture was left without irradiation and processed in parallel for TEM. Various ultrastructural profiles were defined: (I) EL, (II) ED, (III) initiated cell death, indicated by shrinkage and damage of inner membrane, or (IV) presence of electron-dense (condensation) and translucent (lysis) spots in the bacterial cytosol, (V) dead cells with condense cytosolic materials and severally fragmented or lacking inner membranes, and (VI) the halo-shaped profile with electron densities at peripheries of cytoplasm. **(C)** Comparison of relative numbers of profiles I to V of UV-irradiated and untreated STM WT. VI-type relative number is shown in **Figure 8B**. Number of quantified cells combined from three biological replicates: 716 and 612 for UV-irradiated and non-treated groups, respectively. **(D)** Comparison of accumulated frequency of STM with I and II profiles (ED+EL), to accumulated frequency of STM with morphological impairments (III-V, shrinkage and lysis of various severity). **(E)** Effect of UV irradiation on bacterial survival. Plating of aliquots of STM cultures onto agar plates and CFU determination was performed for all experiments involving UV irradiation. Scale bars, 5 µm **(A, B)**, 500 nm (I-VI).
