# Supplementary figures and images for: Exposure to stressors and antimicrobials induces cell-autonomous ultrastructural heterogeneity of an intracellular bacterial pathogen
Source: Front Cell Infect Microbiol. 2022 Nov 15;12:963354. doi: 10.3389/fcimb.2022.963354 (PMC9705743; doi:10.3389/fcimb.2022.963354)

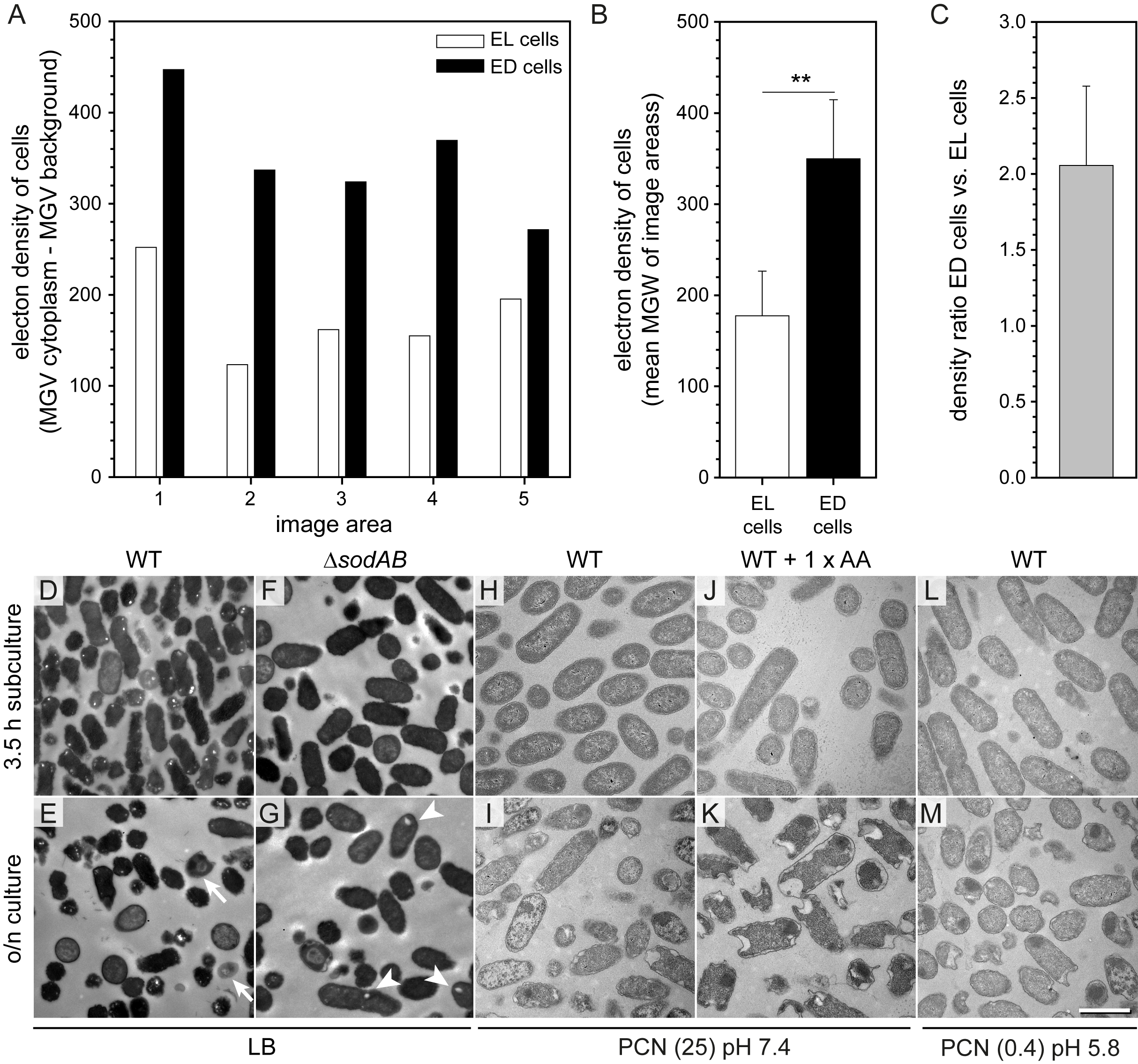

Supplement: Supplementary Figure 1 — (related to Figure 1 ): (A-C) Quantification of electron densities. STM WT or ΔsodAB strains were cultured for 3.5 h or o/n in various medias as indicated and ultrastructure was analysed by 120 keV EF-TEM. (A) Comparison of electron densities (difference between MGV of STM cytoplasm and MGV of background) of ED and EL STM WT in various regions of specimen. Averages of electron densities (B), and averaged density ratio (C) (mean ± SD). (D–G) Electron micrographs were obtained for STM WT and ΔsodAB strains from o/n cultures or 3.5 h subcultures in LB broth at 50 keV. (H-M), STM WT from o/n or 3.5 h subcultures in PCN, pH 7.4 with (J, K), or without AA supplementation (H, I), or in PCN, pH 5.8 (L, M). Arrows in E indicate cells with mixed electron densities and halo-shaped distribution. Arrowheads in G indicate translucent spots in STM ΔsodAB cells of o/n culture not found in 3.5 h subculture. Scale bar, 1 µm. Data are combined from three biological replicates (A–C) and representative micrographs are shown (D–M). Statistical analysis was accomplished by Student’s t-test, and significance levels are indicated by **, p < 0.01. [file Image_1.tif]

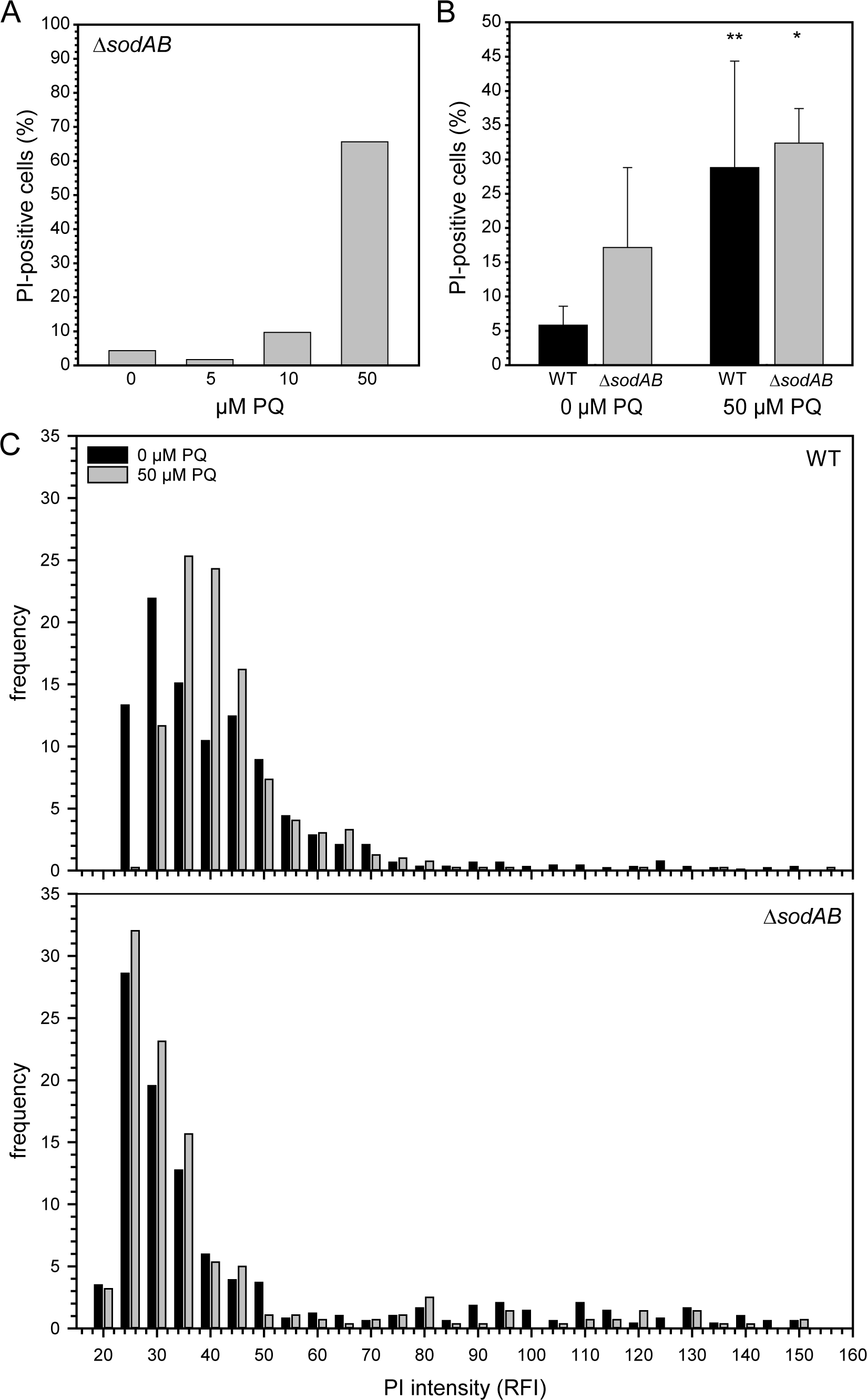

Supplement: Supplementary Figure 2 — (related to Figure 2 ): Paraquat treatment affects membrane integrity to similar extend in STM WT and ΔsodAB strains. (A) Relative numbers of PI-positive STM ΔsodAB without and with treatment with paraquat (PQ). Quantification of PI-positive cells in cultures after treatment with 5, 10 or 50 µM PQ was achieved in comparison to control cultures (no treatment). Number of cells quantified combined from three biological replicates: 3,876, 1,5825, 15,804, and 18,016, for 0 µM, 5 µM, 10 µM, and 50 µM PQ, respectively. (B, C) Effects of treatment of STM WT and ΔsodAB without or with 50 µM PQ on PI fluorescence intensities. The relative number of PI-positive cells and fluorescence intensities of PI. PI was added 12 h after PQ treatment. STM ΔsodAB shows high variability in number and degree (intensities) of PI-positive cells. Statistical significance between controls and PQ-treated cells was calculated by Student’s t-test and significance levels are indicated as *, p < 0.05; **, p < 0.01. [file Image_2.tif]

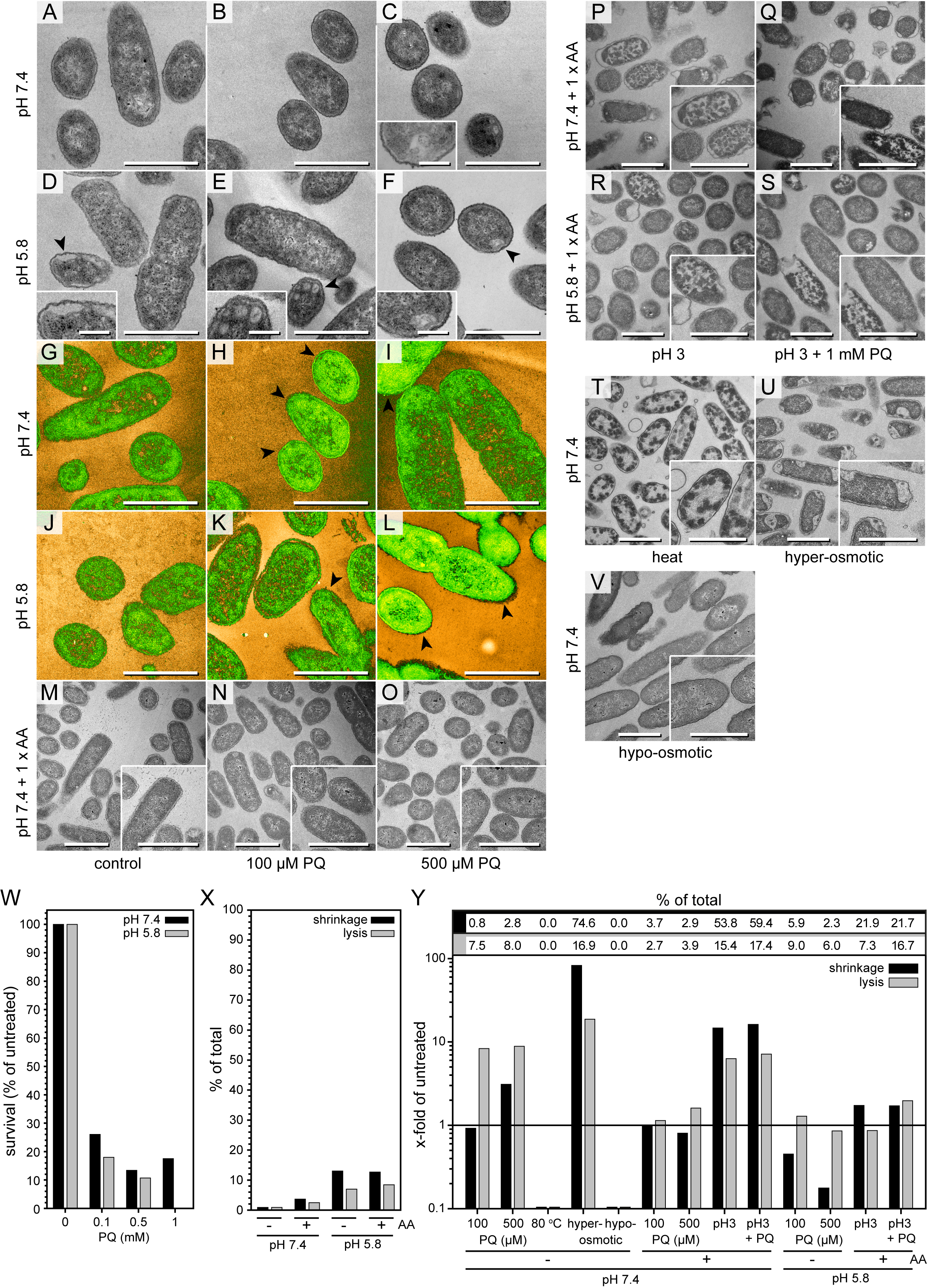

Supplement: Supplementary Figure 3 — (related to Figure 3 ): Treatment of STM WT with various stressors has impact on ultrastructure. (A–F) Electron micrograph of STM WT cultured in PCN, pH 7.4 for 3.5 h and shifted to fresh PCN, pH 7.4 (A–C), or PCN, pH 5.8 (D–F) for incubation without PQ (control A, D), with 100 µM PQ (B, D-E), or 500 µM PQ (C, F). (G–L) EF-TEM micrographs of STM WT without (control), after 100 µM or 500 µM PQ treatment, displayed as an electron density scale. Arrowheads point to cells with denser cytoplasm compared to controls. (M–O) EF-TEM micrographs of STM WT cultured in PCN, pH 7.4 supplemented with AA for 3.5 h and shifted to fresh PCN, pH 7.4 for incubation without (control M), with 100 µM PQ (N), or 500 µM PQ (O). (P–S) EF-TEM micrographs of STM WT cultured in PCN, pH 7.4 or PCN, pH 5.8 supplemented with AA for 3.5 h and shifted to fresh PCN, pH 3.0 for incubation without (P, R), or with 1 mM PQ (Q, S). (T–V) EF-TEM micrographs of STM WT cultured in PCN, pH 7.4 for 3.5 h and shifted to fresh PCN, pH 7.4 for incubation at 80°C (T), to PCN, pH 7.4 containing 600 mM NaCl (U), or to pure H2Odd (V). (W) Aliquots of STM subcultured for 3.5 h in PCN, pH 7.4 or PCN, pH 5.8 without PQ treatment, or treatment with 100 µM, 500 µM or 1 mM PQ were plated onto agar plates, and CFU were determined. Bacterial survival is expressed as percentage of untreated culture. (X, Y) Relative numbers of STM WT with shrinkage (arrowhead in D) or features of lysis (arrowhead in F) in indicated culture conditions. Features of untreated control samples in % of total are shown in (X) and x-fold increase of features of stressor-treated samples compared to the respective untreated sample is shown in (Y). In addition, values of shrinkage and lysis of stress-treated samples in % of the total population is indicated above. 100-300 cells were quantified cells for each condition, combined from three biological replicates. Scale bars, 1 µm (A–V), 250 nm (detail in C–F). [file Image_3.tif]

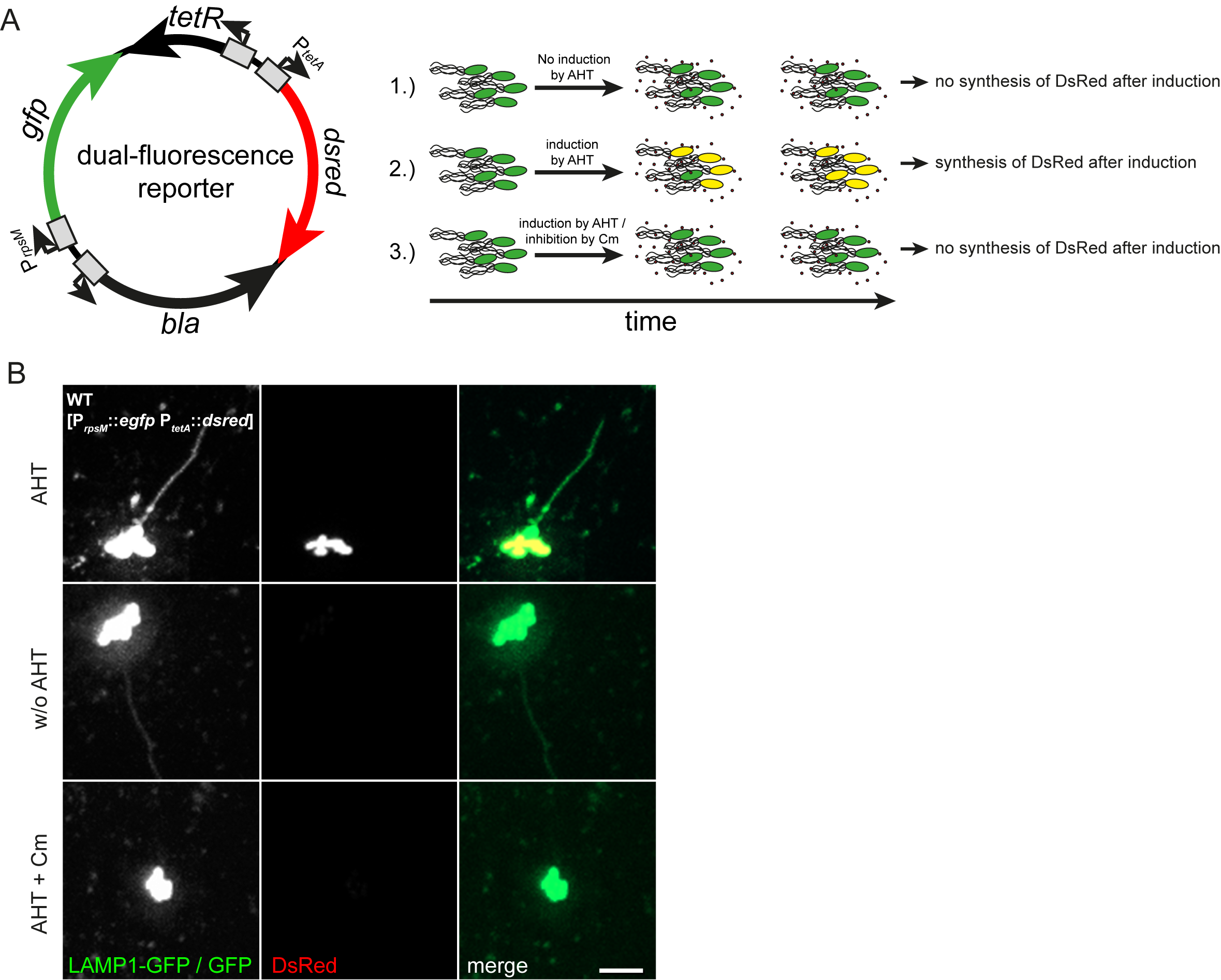

Supplement: Supplementary Figure 4 — (related to Figure 5 - 7 ): Functional characterization of dual-fluorescence reporter in STM WT. (A) The dual-fluorescence reporter harbors cassettes for constitutive gfp expression, and AHT-inducible expression of dsred. Metabolically active bacteria are expected to synthesize DsRed after addition of AHT, in contrast to metabolically inactive bacteria, or if protein biosynthesis is experimentally blocked by chloramphenicol (Cm) after induction. (B) Live cell imaging of intracellular STM WT with dual-fluorescence reporter (GFP) in HeLa cells 8 h p.i. Bacteria were DsRed positive (yellow in merge) only after addition of AHT. AHT was added 4 h p.i. Addition of Cm with AHT blocks DsRed synthesis. Scale bar, 5 µm. [file Image_4.tif]

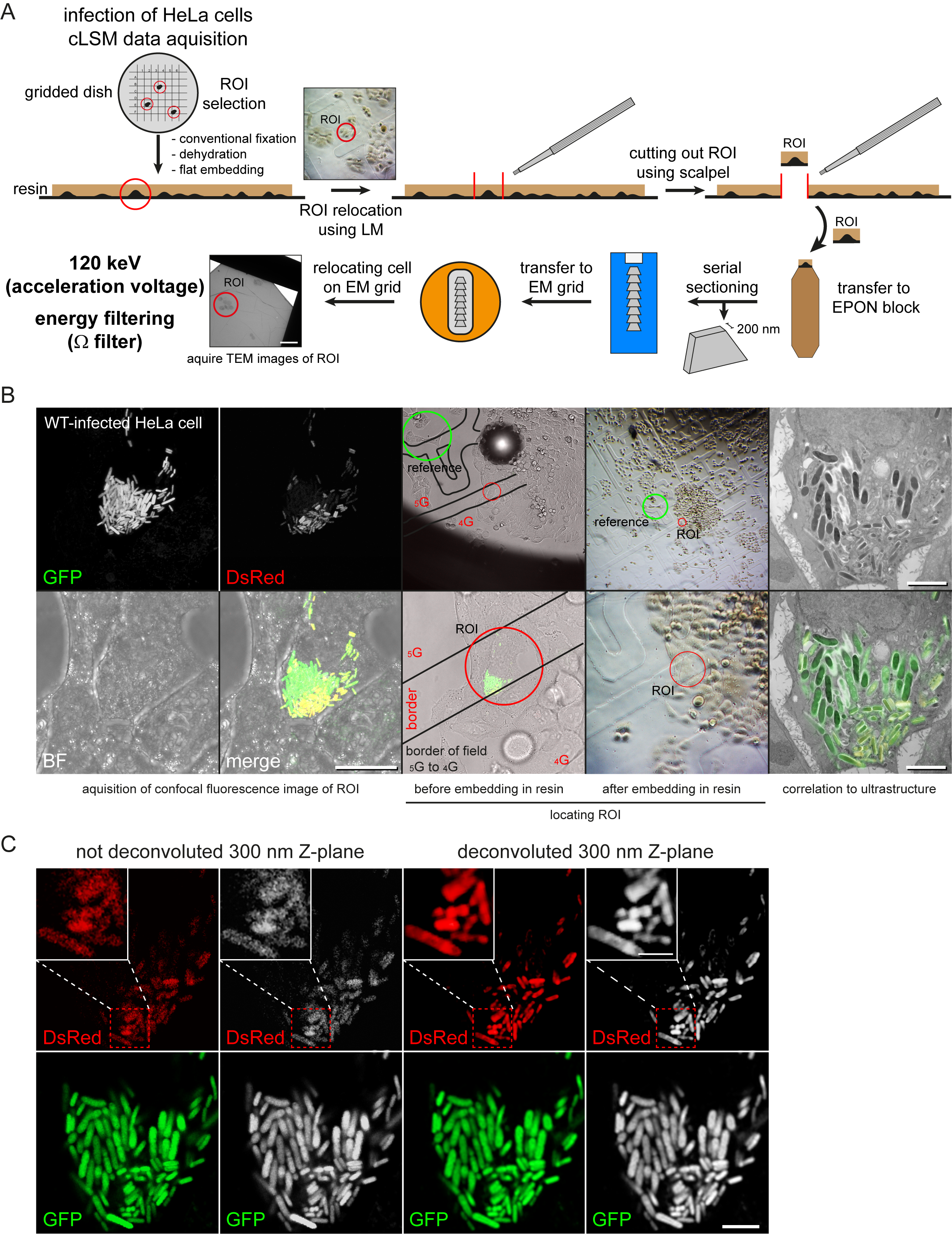

Supplement: Supplementary Figure 5 — (related to and Figure 6 , 7 ): CLEM approach to analyze intracellular STM at high resolution. (A) To register coordinates for CLEM, HeLa cells were seeded at a gridded cover slip prior to infection with STM WT harboring the dual-fluorescence reporter. Gfp was constitutively expressed in STM WT, while dsred expression was induced by addition of AHT 2 h prior to imaging of selected ROIs by CLSM at 16 p.i. Subsequently, samples were fixed and prepared for TEM by flat-embedding in plastic resin. Coordinates from CLSM were well visible, allowing relocation of ROIs. Resin fragments containing ROIs were dissected and individually fixed to resin blocks for serial 200 nm ultra-sectioning. After relocation of HeLa cells containing STM WT in TEM modality, serial sections were acquired at 120 keV with energy filtering (Ω filter). This approach significantly reduced time needed for sample preparation to image whole cells with TEM, providing the opportunity to collect data of high quality at optimal resolutions for correlation or TEM analysis. (B) Examples of the CLEM workflow: visualization by CLSM of intracellular STM WT by virtue of GFP fluorescence, and evaluation of metabolic activity by virtue of DsRed intensity (Z-stack through STM population, maximum intensity projection is shown), registration of positions of infected HeLa cells using BF-LM, relocalization of HeLa cells in resin using imprinted CLEM coordinates, TEM imaging, correlation of single CLSM planes with corresponding single TEM images. (C) Comparison of fluorescence signals without and with deconvolution using Huygens software. After deconvolution, location of proteins GFP and DsRed was increased, and signal-to-noise ratio was improved. Scale bars, 20 µm (B, left panel), 5 µm (B, right panel, C), 2 µm (C, detail). [file Image_5.tif]

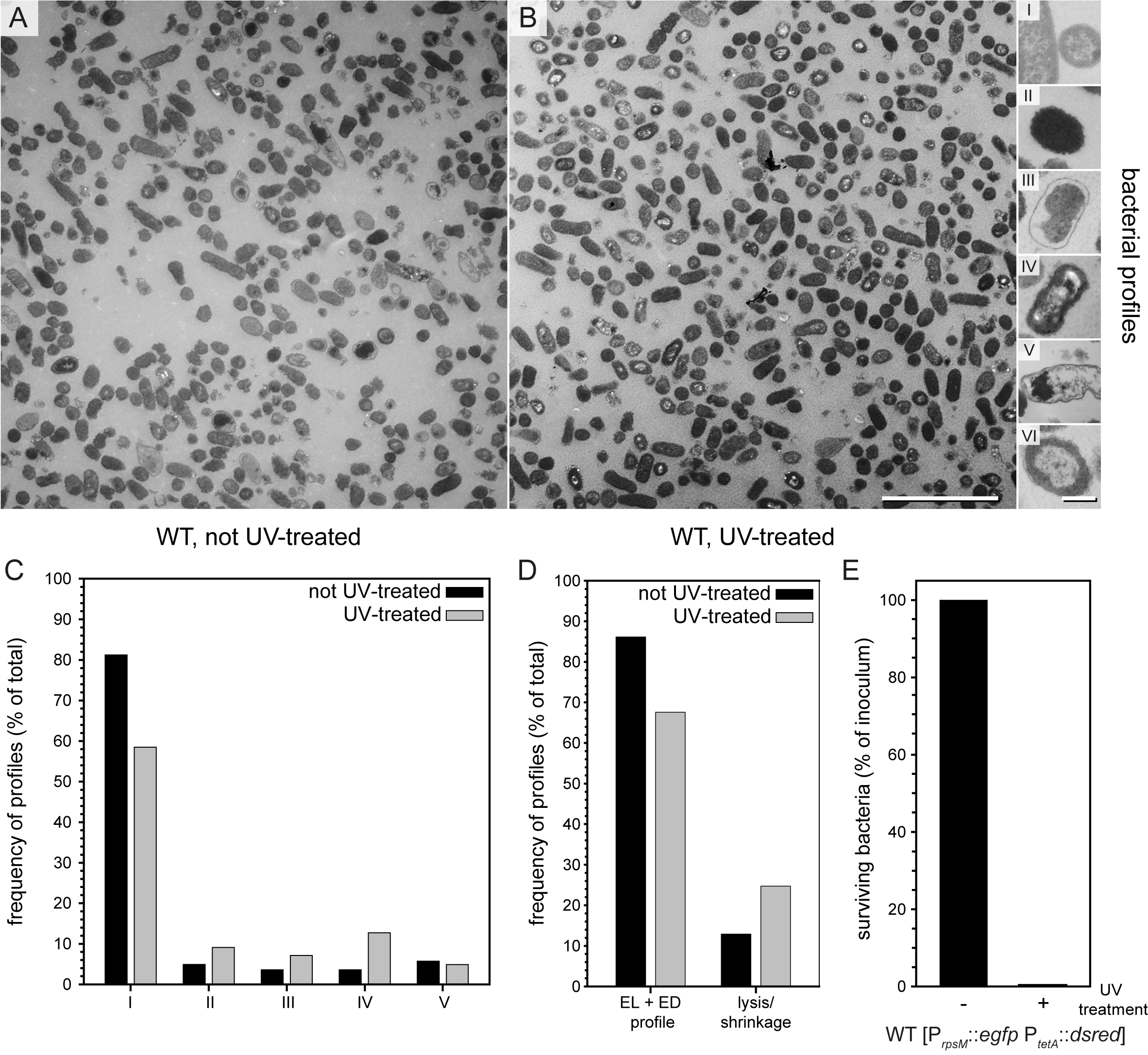

Supplement: Supplementary Figure 6 — (related to Figure 8 ): UV treatment of STM WT increases ultrastructural heterogeneity. (A, B) Cultures of STM WT grown o/n in LB were irradiated 60 sec. with UV light (305 nm) prior preparation for TEM. As control, STM WT of the same culture was left without irradiation and processed in parallel for TEM. Various ultrastructural profiles were defined: (I) EL, (II) ED, (III) initiated cell death, indicated by shrinkage and damage of inner membrane, or (IV) presence of electron-dense (condensation) and translucent (lysis) spots in the bacterial cytosol, (V) dead cells with condense cytosolic materials and severally fragmented or lacking inner membranes, and (VI) the halo-shaped profile with electron densities at peripheries of cytoplasm. (C) Comparison of relative numbers of profiles I to V of UV-irradiated and untreated STM WT. VI-type relative number is shown in Figure 8B. Number of quantified cells combined from three biological replicates: 716 and 612 for UV-irradiated and non-treated groups, respectively. (D) Comparison of accumulated frequency of STM with I and II profiles (ED+EL), to accumulated frequency of STM with morphological impairments (III-V, shrinkage and lysis of various severity). (E) Effect of UV irradiation on bacterial survival. Plating of aliquots of STM cultures onto agar plates and CFU determination was performed for all experiments involving UV irradiation. Scale bars, 5 µm (A, B), 500 nm (I-VI). [file Image_6.tif]
